# Current Technological Advances in Dysphagia Screening: Systematic Scoping Review

**DOI:** 10.2196/65551

**Published:** 2025-05-05

**Authors:** Duo Wai-Chi Wong, Jiao Wang, Sophia Ming-Yan Cheung, Derek Ka-Hei Lai, Armstrong Tat-San Chiu, Dai Pu, James Chung-Wai Cheung, Timothy Chi-Yui Kwok

**Affiliations:** 1 Department of Biomedical Engineering Faculty of Engineering Hong Kong Polytechnic University Hong Kong China (Hong Kong); 2 Department of Clinical Laboratory Hubei Provincial Hospital of Traditional Chinese Medicine Wuhan China; 3 Department of Mathematics School of Science Hong Kong University of Science and Technology Hong Kong China (Hong Kong); 4 Kowloon Home for the Aged Blind Hong Kong Society for the Blind Hong Kong China (Hong Kong); 5 School of Primary and Allied Health Care Monash University Melbourne Australia; 6 Research Institute for Smart Ageing Hong Kong Polytechnic University Hong Kong China (Hong Kong); 7 Department of Medicine and Therapeutics Faculty of Medicine Chinese University of Hong Kong Hong Kong China (Hong Kong); 8 Jockey Club Centre for Positive Ageing Chinese University of Hong Kong Hong Kong China (Hong Kong)

**Keywords:** digital health, computer-aided diagnosis, computational deglutition, machine learning, deep learning, artificial intelligence, AI, swallowing disorder, aspiration

## Abstract

**Background:**

Dysphagia affects more than half of older adults with dementia and is associated with a 10-fold increase in mortality. The development of accessible, objective, and reliable screening tools is crucial for early detection and management.

**Objective:**

This systematic scoping review aimed to (1) examine the current state of the art in artificial intelligence (AI) and sensor-based technologies for dysphagia screening, (2) evaluate the performance of these AI-based screening tools, and (3) assess the methodological quality and rigor of studies on AI-based dysphagia screening tools.

**Methods:**

We conducted a systematic literature search across CINAHL, Embase, PubMed, and Web of Science from inception to July 4, 2024, following the PRISMA-ScR (Preferred Reporting Items for Systematic Reviews and Meta-Analyses extension for Scoping Reviews) framework. In total, 2 independent researchers conducted the search, screening, and data extraction. Eligibility criteria included original studies using sensor-based instruments with AI to identify individuals with dysphagia or unsafe swallow events. We excluded studies on pediatric, infant, or postextubation dysphagia, as well as those using non–sensor-based assessments or diagnostic tools. We used a modified Quality Assessment of Diagnostic Accuracy Studies–2 tool to assess methodological quality, adding a “model” domain for AI-specific evaluation. Data were synthesized narratively.

**Results:**

This review included 24 studies involving 2979 participants (1717 with dysphagia and 1262 controls). In total, 75% (18/24) of the studies focused solely on per-individual classification rather than per–swallow event classification. Acoustic (13/24, 54%) and vibratory (9/24, 38%) signals were the primary modality sources. In total, 25% (6/24) of the studies used multimodal approaches, whereas 75% (18/24) used a single modality. Support vector machine was the most common AI model (15/24, 62%), with deep learning approaches emerging in recent years (3/24, 12%). Performance varied widely—accuracy ranged from 71.2% to 99%, area under the receiver operating characteristic curve ranged from 0.77 to 0.977, and sensitivity ranged from 63.6% to 100%. Multimodal systems generally outperformed unimodal systems. The methodological quality assessment revealed a risk of bias, particularly in patient selection (unclear in 18/24, 75% of the studies), index test (unclear in 23/24, 96% of the studies), and modeling (high risk in 13/24, 54% of the studies). Notably, no studies conducted external validation or domain adaptation testing, raising concerns about real-world applicability.

**Conclusions:**

This review provides a comprehensive overview of technological advancements in AI and sensor-based dysphagia screening. While these developments show promise for continuous long-term tele-swallowing assessments, significant methodological limitations were identified. Future studies can explore how each modality can target specific anatomical regions and manifestations of dysphagia. This detailed understanding of how different modalities address various aspects of dysphagia can significantly benefit multimodal systems, enabling them to better handle the multifaceted nature of dysphagia conditions.

## Introduction

### Background

Dysphagia, a condition characterized by difficulty in swallowing, has been recognized as a significant geriatric syndrome with extensive impacts on the health of older adults and the health care system [1]. This syndrome or disorder is particularly prevalent among older adults with dementia, affecting 58% of this population [2]. The risk of dysphagia is further amplified by some age-related conditions such as Parkinson disease, stroke, postextubation, and other neurogenic or neurodegenerative conditions [3].

The etiology of dysphagia is multifaceted. It could result from disrupted neural pathways leading to poor muscle coordination [4], as well as sarcopenia causing weakness in neck and tongue muscles [5]. Aspiration, leading to aspiration pneumonia, is one of the most severe complications associated with dysphagia. Nearly one-fifth of the patients admitted for aspiration pneumonia are diagnosed with dysphagia [6], and some patients may experience silent aspiration, which does not manifest any obvious signs [7]. The condition is further compounded by its strong association with cognitive disorders [8], making it challenging for individuals with dysphagia to comply with swallowing instructions and training. With a prevalence rate of 60.9% in residential care homes [9], dysphagia significantly impairs the activities of daily living of older adults [10] and increases morbidity [1]. The risk of mortality is particularly high, with patients with dysphagia experiencing a 13-fold higher mortality rate [11]. Moreover, dysphagia is closely linked to malnutrition, with 39.2% of patients at risk [12] and a 4.8 times higher chance of experiencing malnutrition [13].

Early diagnosis of dysphagia is crucial as it facilitates prompt treatment and appropriate management, such as specialized meals with optimized bolus volume and viscosity [14]. Currently, the gold standards for dysphagia diagnosis are instrumental assessments using the videofluoroscopic swallowing study (VFSS) or fiberoptic endoscopic evaluation of swallowing (FEES). However, these methods pose risks to patients, require trained personnel, have limited accessibility, and are unsuitable for routine screening [1,15]. Therefore, there is a heavy reliance on noninstrumental bedside screening methods, including clinical tests and questionnaires. The Eating Assessment Tool is one of the most common screening questionnaires, with a relatively low specificity of 0.59 [16]. Several surveys on dysphagia have been devised using self-report questionnaires, including the Dysphagia Risk Assessment for the Community-Dwelling Elderly [17], Sydney Swallow Questionnaire [18], and Ohkuma questionnaire for dysphagia [19]. However, these instruments have not demonstrated sufficient quality of evidence regarding their psychometric properties when used with older adults [20]. Clinical tests, such as water swallowing tests and the Gugging Swallowing Screen, demonstrate wide variability in sensitivity and specificity [21-23]. These accessible bedside methods remain subjective and examiner dependent, potentially underestimating dysphagia incidence. One report suggested that questionnaire-based assessments may miss up to half of dysphagia cases [9]. These limitations could be particularly problematic in residential care homes, where dysphagia is often underdiagnosed and undertreated due to resource constraints, lack of routine screening [24], and limited staff awareness [25]. Given these challenges, there is a pragmatic demand for more accessible, objective, and reliable screening tools that can be easily implemented, especially in residential care home settings, to improve early detection and management.

The emergence of advanced technologies, including sensors, computer vision, and artificial intelligence (AI), has opened up new avenues for the screening and diagnosis of dysphagia that address some limitations regarding cost, accessibility, and the need for specialized personnel despite the fact that their performance has shown variability and potential bias [26]. However, a systematic review demonstrated a pooled diagnostic odds ratio of 21.5 for wearable technology in identifying aspiration, highlighting the potential of these devices to enhance clinical detection of aspiration [27]. AI models, including machine learning and deep learning, are increasingly being integrated into dysphagia screening tools [26,27], especially in the application of computer vision. Lai et al [28] used depth video data and combined them with transformer models and convolutional networks to classify swallowing tasks, whereas Yamamoto et al [29] used a Kinect 3D camera to quantify swallowing dynamics during bolus flow. In addition, soft sensors or electronic skin incorporating materials such as carbon nanotubes [30] and graphene [31] have shown potential in monitoring swallowing activities. These technological advancements, referred to as computer-aided dysphagia screening or computational deglutition, hold significant promise for improving early detection. As dysphagia is a gradual process, the integration of these technologies to tackle the accessibility issue could facilitate more frequent screening, thereby enabling earlier detection.

### Objectives

To this end, the objective of this review was to explore the current state-of-the-art AI-based instruments for dysphagia screening. Specifically, this review will address the following questions: (1) what are the current AI-based instruments and their protocols for dysphagia screening? (2) How well do these AI-based instrument screening tools perform? (3) Are the studies on AI-based dysphagia screening tools well reported and methodologically rigorous?

## Methods

### Eligibility Criteria

We followed the PRISMA-ScR (Preferred Reporting Items for Systematic Reviews and Meta-Analyses extension for Scoping Reviews) [32] in the reporting of this study (Multimedia Appendix 1). The search strategy was developed through the collaborative effort of a multidisciplinary team comprising experts from relevant fields, including biostatistics (DWCW), occupational therapy (ATSC), speech therapy (DP), biomedical engineering (JCWC), and geriatric medicine (TCYK). We included original studies that identified individuals with dysphagia, unsafe swallows from individuals without dysphagia, or safe swallows using sensor-based instruments with AI models. We focused on AI models designed for prediction. These models are trained on datasets and then tested on separate data to ensure their accuracy. This testing is done either by splitting the data into training and testing sets or through a process called cross-validation. They may include advanced statistical models, traditional machine learning models, deep learning models, or generative models. In addition, to be included, studies must involve experiments or data sourced from human participants. While some level of data augmentation or use of generative data was acceptable, the foundation of the data must be derived from human participants.

Studies on pediatric or infant dysphagia, as well as dysphagia secondary to postextubation, would be excluded as these forms of dysphagia have different considerations. In addition, studies would be excluded if they focused on non–sensor-based bedside clinical assessments, questionnaires, and prediction models based on clinical data, as well as diagnostic tools such as the VFSS, the FEES, manometry, and other medical imaging.

### Search Strategy and Selection Criteria

The literature search was conducted on several databases: CINAHL (via EBSCOhost), Embase (via Ovid), PubMed, and Web of Science. The search included academic journal papers (preprint and in-press inclusive) and full conference papers from the inception of each database to the current date (July 4, 2024). We limited our search to publications in English without restricting the search by country of origin or publication source.

The literature search was conducted using a combination of keywords related to 4 main domains: participants (ie, dysphagia), models, instruments, and outcomes. To refine the results, exclusion terms were applied using the *NOT* operator to filter out studies on pediatric, infant, or postextubation dysphagia. The full search terms and queries are provided in Multimedia Appendix 2. The literature search, initial screening, and eligibility assessment were independently carried out by 2 researchers (DWCW and JW). Any disagreements were resolved through consensus with the corresponding author or, when necessary, other coauthors. Duplicates were initially removed using the automatic duplication detection function in the citation and reference management tool EndNote (version 20; Clarivate Analytics). The remaining entries were manually checked by 2 researchers (SMYC and DKHL) to remove any missed duplicates. The initial screening was conducted by reviewing the titles, abstracts, and keywords, whereas the eligibility assessment was conducted by examining the full texts. In addition, a snowball search was conducted on the reference lists of eligible articles to uncover any potentially overlooked studies. The references were managed using EndNote.

### Data Extraction and Synthesis

For data extraction, we proposed a modified thematic framework based on the traditional participants, index test, reference test, and outcome model. Our adapted framework consisted of 5 main themes: participants, modalities, protocols, models, and performance, which is more technologically oriented. In the modified theme, the reference test component was subsumed under the *participants* theme, whereas the protocols were incorporated into the *modalities* theme. We used a Sankey diagram to illustrate the relationship and mappings among various components across different themes. For the *performance* theme, we identified and extracted 5 common metrics used for evaluating model performance. These were accuracy, *F*_1_-score, area under the receiver operating characteristic curve (AUC), sensitivity, and specificity. We presented the models (per individual class) that performed the best based on *F*_1_-score followed by accuracy and sensitivity.

To evaluate the methodological quality of the included studies, we used a modified version of the Quality Assessment of Diagnostic Accuracy Studies–2 (QUADAS-2) tool [33], dubbed QUADAS-2+M. The original instrument assesses 4 key domains: patient selection, index test, reference standard, and flow and timing in terms of the risk of bias and applicability. To address the specific needs of evaluating AI-based diagnostic studies, we proposed an additional domain focused on the AI model inspired by the Transparent Reporting of a Multivariate Prediction Model for Individual Prognosis or Diagnosis + Artificial Intelligence (TRIPOD+AI) guidelines [34]. The fifth domain (*model*), as shown in Textbox 1, included signaling questions on risk of bias covering the aspects of hyperparameter tuning, handling of class imbalance, and missing data. The applicability assessment focused on the models’ transferability, specifically evaluating whether the model’s performance was adaptable when deployed. We presented the assessment results for the model domain separately from those of the original QUADAS-2.

**Quality Assessment of Diagnostic Accuracy Studies–2 dubbed assessment domain and signaling questions with expanded criteria including the model domain.**

**Risk of bias—is there a possibility that the model’s development, training, or testing processes could have introduced bias?**

- Signaling question 1: Was the model subjected to hyperparameter tuning, if applicable?
- Signaling question 2: If a significant class imbalance exists, were any measures taken to address it?
- Signaling question 3: If any data were missing, were appropriate methods used to handle them during model training?

**Applicability—are there concerns regarding the model’s applicability in real-world scenarios?**

- Signaling question: Were any attempts made to demonstrate the model’s applicability or generalizability through external testing, domain adaptation, robust testing, or other methods?

## Results

### Search Results

**Figure 1:**
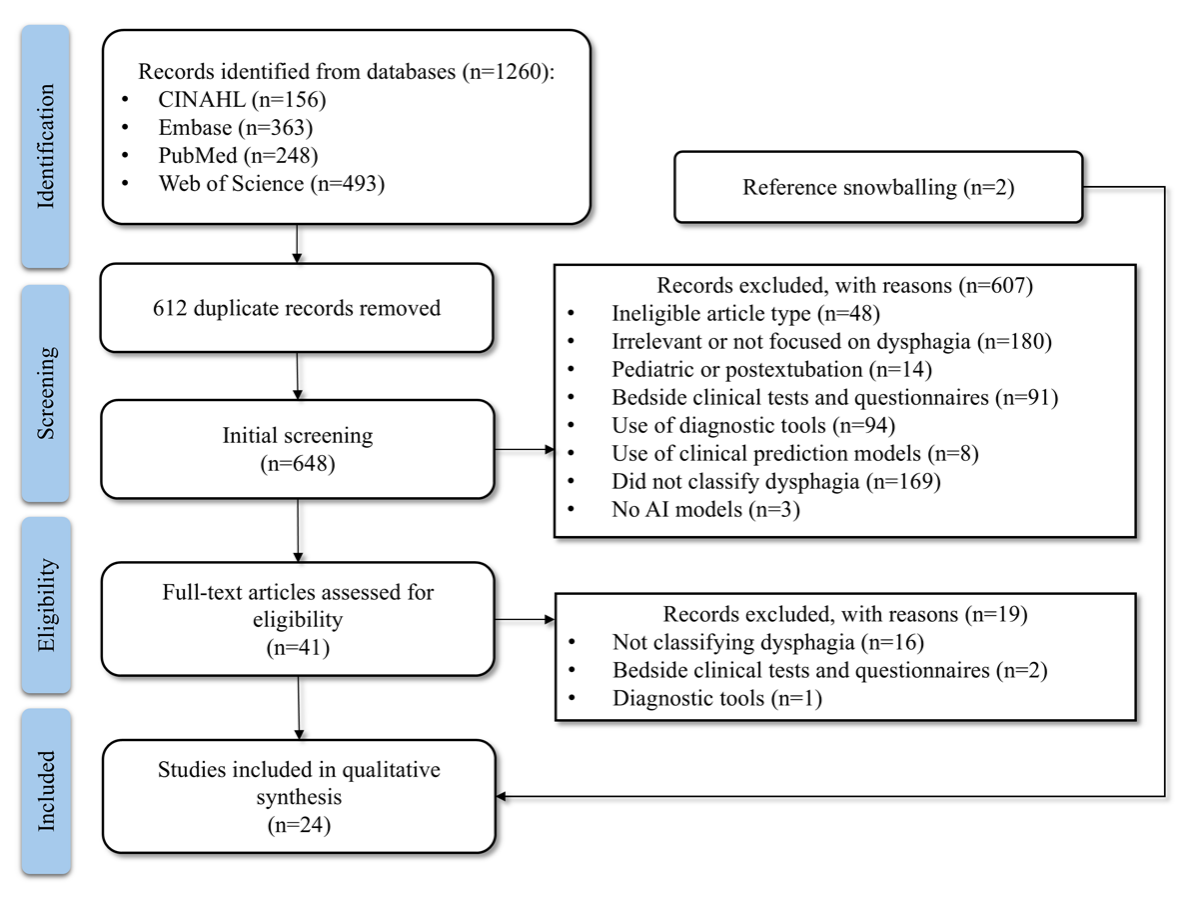
PRISMA (Preferred Reporting Items for Systematic Reviews and Meta-Analyses) flowchart of the systematic search and screening. This diagram illustrates the systematic literature search and screening and the number of eligible articles for review. AI: artificial intelligence.

**Figure 2:**
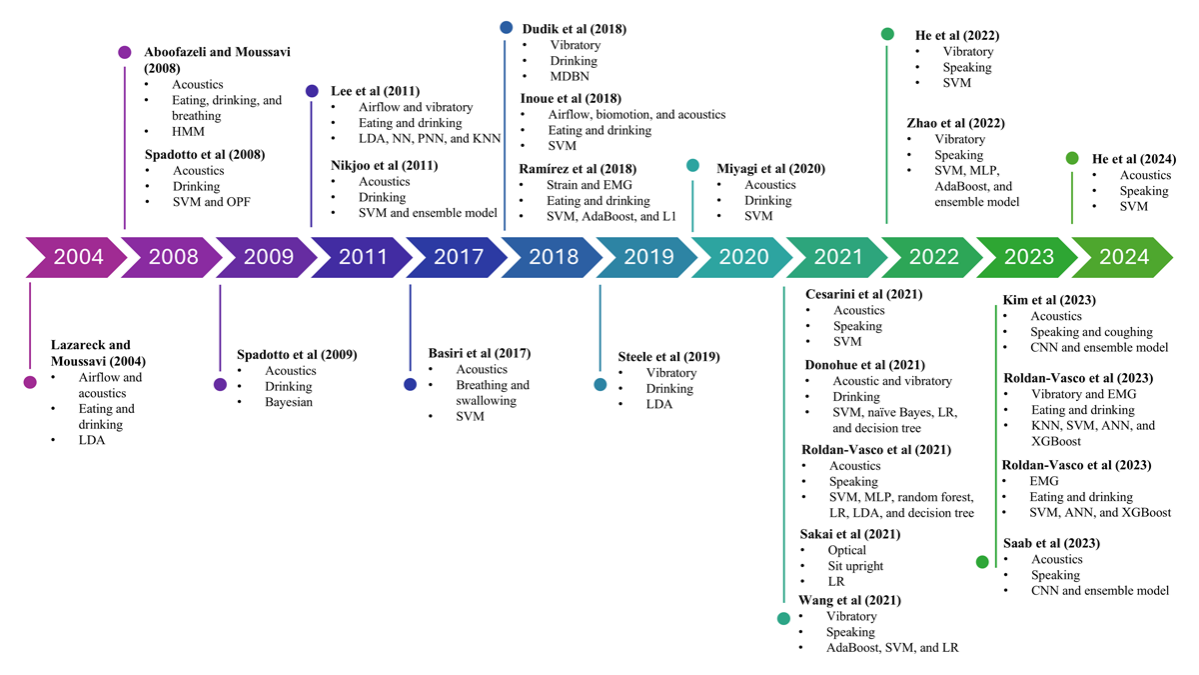
Timeline of publications on technological advancements for dysphagia screening [35-58]. AdaBoost: Adaptive Boosting; ANN: artificial neural network; CNN: convolutional neural network; EMG: electromyography; HMM: hidden Markov model; KNN: k-nearest neighbor; L1: Lasso regularization; LDA: linear discriminant analysis; LR: logistic regression; MDBN: multilayer deep belief network; MLP: multilayer perceptron; NN: neural network; OPF: optimum-path forest; PNN: probabilistic neural network; SVM: support vector machine; XGBoost: Extreme Gradient Boosting.

As shown in Figure 1, the initial search yielded 1260 entries (Multimedia Appendix 3), of which 648 (51.43%) proceeded to preliminary abstract screening after duplicates were removed. Initial screening based on title, abstract, and keywords excluded 607 articles based on the following criteria: ineligible article types (eg, review, commentary, and protocol papers; n=48, 7.9%), irrelevance of lack of focus on dysphagia investigations (n=180, 29.7%), studies on pediatric or postextubation-induced dysphagia as per the exclusion criteria (n=14, 2.3%), focus on bedside clinical tests and questionnaires (n=91, 15%), use of diagnostic tools (n=94, 15.5%) and clinical prediction models (n=8, 1.3%), studies not aiming at the classification of dysphagia and nondysphagia (n=169, 27.8%), and absence of AI models (n=3, 0.5%). Screening of the full texts for eligibility further excluded 19 articles, with reasons including not aiming at the classification of dysphagia and nondysphagia (n=16, 84%), focus on bedside clinical tests and questionnaires (n=2, 11%), and use of diagnostic tools (n=1, 5%). A total of 2 studies were added from snowballing the references of the eligible articles. Eventually, there were 24 articles eligible for this review [35-58]. The eligible studies are shown in the timeline graph in Figure 2 [35-58].

A total of 12% (3/24) of the studies conducted classification at both the individual and episode levels [36,44,48]. In other words, they distinguished between individuals with dysphagia (at risk) and without dysphagia, as well as between safe and unsafe swallowing events. Another set of 12% (3/24) of the studies focused on classifying safe and unsafe swallows among individuals with dysphagia [45,47,56]. The remaining studies (18/24, 75%) conducted the classification exclusively on a per-individual basis.

### Methodological Quality Assessment

Several discussions were held regarding inconsistencies in the grading approach. We reached the following consensus and made the following notes. To assess the risk of bias in patient selection, the signaling questions focused on whether consecutive or random samples were enrolled. We assigned an “unclear” grade if the study did not address the sampling methods, which was the case in most instances. To assess the risk of bias in the index test, studies were graded as “unclear” if they did not specify whether blinding was conducted. For the reference test, we assigned an “unclear” grade in cases in which screening tests were applied for making diagnoses or in which the study did not describe how diagnoses were conducted.

**Figure 3:**
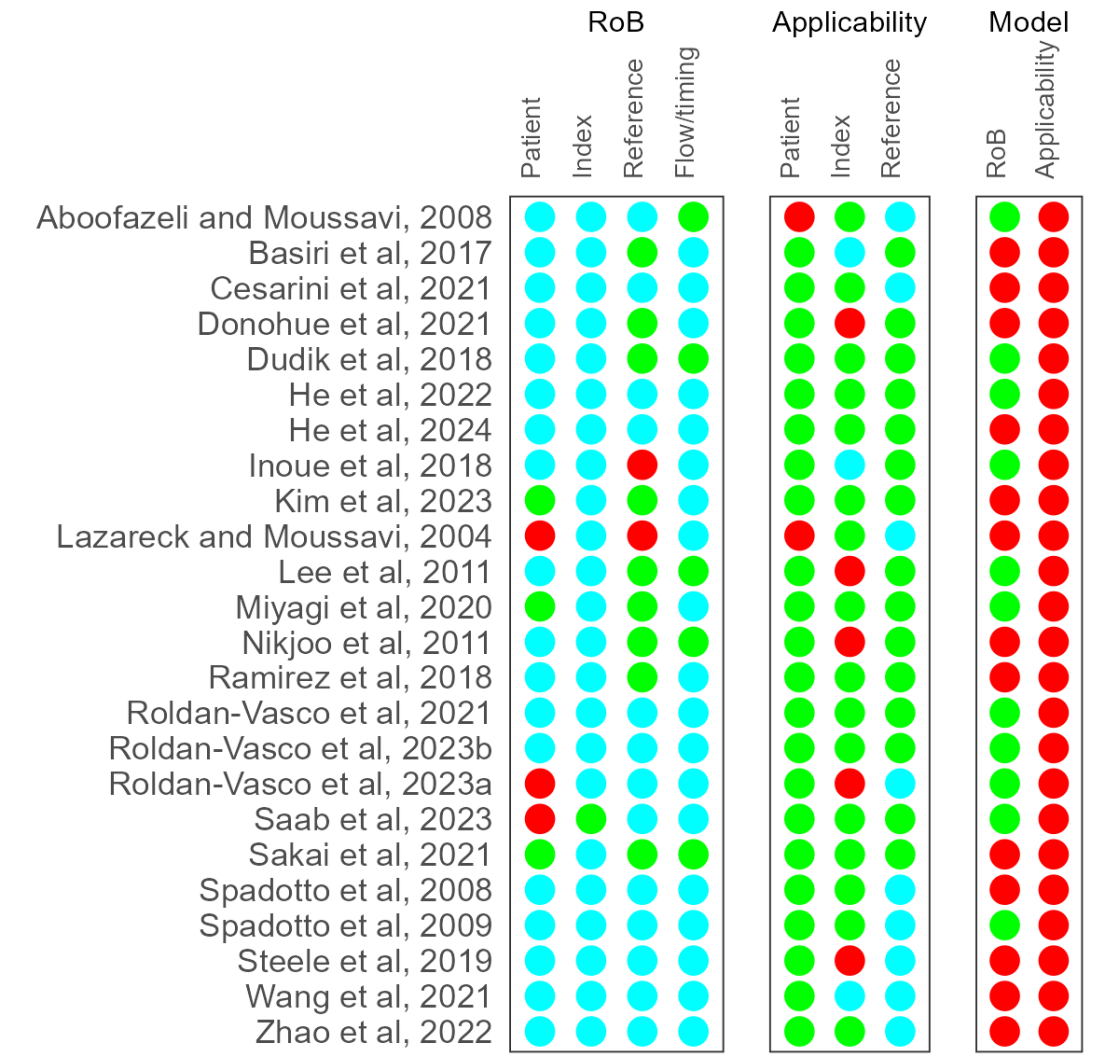
Assessment results of the modified Quality Assessment of Diagnostic Accuracy Studies–2 with an additional model domain [35-58]. Green indicates low risk, red indicates high risk, and cyan indicates unclear risk. RoB: risk of bias.

As shown in Figure 3 [35-58], most of the studies exhibited an unclear risk of bias, including in patient selection (18/24, 75%), index test (23/24, 96%), reference standard (13/24, 54%), and flow and timing (19/24, 79%). This was primarily because many studies (18/24, 75%) did not describe their sampling approaches or mention whether they had blinded the labels of the case-control groups. In addition, studies reported enrolling dysphagia cases but did not explain how these cases were confirmed. While some studies (4/24, 17%) conducted the VFSS concurrently with the index test (ie, flow and timing), they indicated that the VFSS was used to segment the signal, leaving it unclear whether it was also used to reconfirm the dysphagia status.

Satisfactory results in the applicability of patient selection (22/24, 92%), index test (16/24, 67%), and reference standard (15/24, 62%) were obtained. These studies were case-control, and we assumed that the diagnoses were confirmed. However, some studies received high (5/24, 21%) or unclear (3/24, 12%) risk ratings for the index test because the protocols for conducting the screening differed between participants with and without dysphagia. This discrepancy arose primarily because, for tasks such as eating, researchers needed to control the consistency and volume of food to ensure safety for the participants with dysphagia.

In the new *model* domain, illustrated in the 2 rightmost columns in Figure 3 [35-58], 54% (13/24) of the studies showed a high risk of bias, and no study satisfied the applicability criterion. This bias may be due to unclear or inadequate hyperparameter tuning (12/24, 50%) or methods for addressing significant class imbalance (7/24, 29%) whether between groups (dysphagia vs nondysphagia), episodes (safe swallows vs unsafe swallows), or different kinds of swallow or nonswallow tasks. Notably, none of the studies conducted external testing or domain adaptation testing, which significantly impacts the transferability and applicability of the system.

### Participant Characteristics

The 24 studies in this review included a total of 2979 participants: 877 male, 1054 female, and 1048 individuals whose sex was not specified (Table 1). Of these participants, 1717 were individuals with dysphagia (confirmed, suspected, or at risk), whereas 1262 were healthy controls without dysphagia.

In addition to the initial clinical diagnoses of dysphagia, 62% (15/24) of the studies conducted additional reference tests to confirm the dysphagia status, some of which were conducted concurrently with the device or technology being evaluated. Of these 15 studies, 11 (73%) used diagnostic tools such as the VFSS and FEES, whereas 5 (33%) used bedside clinical assessments or questionnaires. Using the VFSS, Lee et al [45] assessed dysphagia through a 4-point bolus clearance scale measuring the depth of airway invasion and bolus clearance from the valleculae and the pyriform sinuses and established 3 sets of “ground truths” indicating the presence of dysphagia. Conversely, Saab et al [52] characterized the “true” presence of dysphagic condition through an evaluation conducted by a trained examiner using the Toronto Bedside Swallowing Screening Test [59]. This test included tasks to assess changes in voice, evaluate repetitive swallowing, and screen for dysphonia.

**Figure 4:**
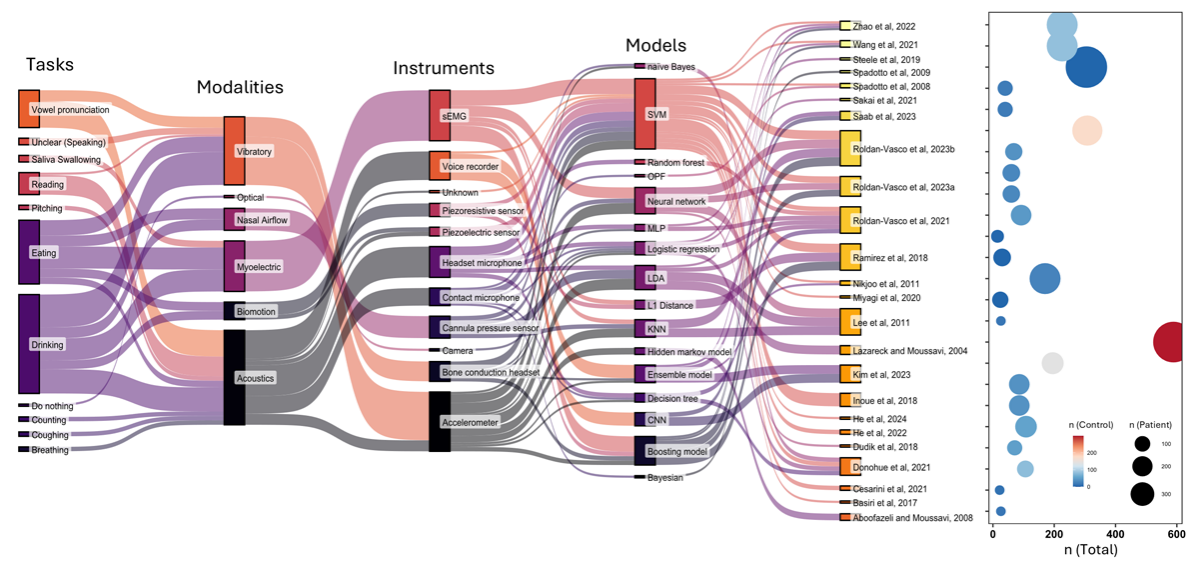
Sankey diagram illustrating the mapping of key context of the reviewed papers and the study sample sizes [35-58]. CNN: convolutional neural network; KNN: k-nearest neighbor; LDA: linear discriminant analysis; MLP: multilayer perceptron; OPF: optimum-path forest; sEMG: surface electromyography; SVM: support vector machine.

The mean age of the participants varied across the studies. In the studies that provided this information, the mean age ranged from 41.23 to 86.22 years in the dysphagia group and from 22.4 to 83.3 years in the nondysphagia group. Canada (6/24, 25%) and China (4/24, 17%) were the predominant sources of the studies, followed by the United States, Japan, and Colombia (3/24, 12% each). Other countries included Brazil (2/24, 8%) as well as Iran, Italy, Republic of Korea, and Spain (1/24, 4% each). Figure 4 [35-58] shows a Sankey diagram that illustrates the number of participants in each study and the evidence mapping toward modality, protocol, and model.

**Table 1:** Demographic information of the participants in the included studies.

Study	Dysphagia (or suspected)		Controls without dysphagia (healthy)		Remarks	Reference test^a^
	Participants, n	Details	Participants, n	Details		
Aboofazeli and Moussavi [35]	11	Aged 16-25 y	15	12 children (aged 3-16 y) and 3 healthy adults (aged 35, 38, and 54 y)	—^b^	VFSS^c,d^
Basiri et al [36]	11	Number of swallowing episodes: 108; GERD^e^	11	Per swallowing episodes: 116	Pooled: 12 male and 10 female participants aged 21-76 y; both per individual and per episode	—
Cesarini et al [37]	26	13 male and 13 female	80	40 male and 40 female	—	—
Donohue et al [38]	20	10 male and 10 female; mean age 61.25 (range 35-82) y; ND^f^	51	22 male and 29 female; mean age 67.21 (range 39-87) y	—	VFSS
Dudik et al [39]	53	34 male and 19 female; mean age 63 y; per swallowing episodes: 963	55	28 male and 27 female; mean age 39 y; per swallowing episodes: 1650	—	VFSS
He et al [40]	46	20 male and 26 female; mean age 84.3 (SD 5.45) y	40	17 male and 23 female; mean age 82.6 (SD 6.98) y	—	WST^g^ and EAT-10^h^
He et al [41]	46	20 male and 26 female; mean age 84.3 (SD 5.45) y	40	17 male and 23 female; mean age 82.6 (SD 6.98) y	—	WST and EAT-10
Inoue et al [42]	55^i^	18 male and 37 female; mean age 75.5 (SD 20.5; range 60-99) y; per swallowing episodes: 288	140	57 male and 83 female; mean age 54.5 (SD 32.5; range 20-89) y; per swallowing episodes: 1241	—	WST, MWST^j^, and RSWT^k^
Kim et al [43]	290	Mean age 68.8 (SD 12.6) y	299	Mean age 60.8 (SD 14.5) y	Pooled age: mean 60.8 (SD 14.5) y	VFSS
Lazareck and Moussavi [44]	11	Aged 16-25 y	15	12 children (aged 3-16 y) and 3 healthy adults (aged 35, 38, and 54 y)	Both per individual and per episode	VFSS
Lee et al [45]	24	22 male and 2 female; mean age 64.8 (SD 18.6) y; stroke or ABI^l^	—	—	Classification per episode	VFSS, x-ray, and bolus clearance scale
Miyagi et al [46]	143	78 male and 65 female; mean age 83.3 (range 25-102) y	27	17 male and 10 female; mean age 22.4 (range 21-47) y	—	FEES^m^
Nikjoo et al [47]	30	15 male and 15 female; mean age 65.47 (SD 13.4) y; NG^n^	—	—	Classification per episode; safe (n=60) vs unsafe (n=164) swallows	VFSS
Ramírez et al [48]	14	12 male and 2 female; aged 43-83 y; head-neck cancer	1	1 male; aged 24 y	Both per individual and per episode	VFSS
Roldan-Vasco et al [49]	46	23 male and 23 female; mean age 60.04 (SD 12.37) y; NG and NM^o^	46	23 male and 23 female; mean age 60.17 (SD 11.93) y	—	—
Roldan-Vasco et al [50]	30	15 male and 15 female; mean age 41.23 (SD 14.45) y; NG and NM	30	15 male and 15 female; mean age 39.10 (SD 15.05) y	—	—
Roldan-Vasco et al [51]	29	16 male and 13 female; mean age 45.69 (SD 11.92) y; NG and NM	31	17 male and 14 female; mean age 45.29 (SD 16.22) y	—	—
Saab et al [52]	28	19 male and 9 female; mean age 69 (SD 17) y for training and 73 (SD 18) y for testing; stroke	40	16 male and 24 female; mean age 67 (SD 16) y for training and 65 (SD 16) y for testing; stroke	—	TOR-BSST^p^
Sakai et al [53]	133	61 male and 72 female; mean age 86.22 (SD 7.47) y; sarcopenia	175	67 male and 108 female; mean age 82.57 (SD 8.01) y	—	MWST and VFSS
Spadotto et al [54]	20	NG	20	—	—	—
Spadotto et al [55]	20	After CVA^q^	20	—	—	—
Steele et al [56]	305	Stroke or ABI	—	—	Before dropout: 167 male and 167 female; mean age 72 y; classification per episode	VFSS
Wang et al [57]	143	47 male and 96 female; mean age 84.7 (SD 5.6) y	83	35 male and 48 female; mean age 83.3 (SD 5.3) y	—	—
Zhao et al [58]	143	47 male and 96 female; mean age 84.7 (SD 5.6) y	83	35 male and 48 female; mean age 83.3 (SD 5.3) y	—	—

^a^Reference test in this context refers to the procedure used to confirm dysphagia in addition to the initial clinical diagnosis or history of medical diagnosis.

^b^No remarks.

^c^VFSS: videofluoroscopic swallowing study.

^d^The studies used VFSS for segmentation during the experiment, but it was not explicitly stated whether it was used to reconfirm the disease state (reference test).

^e^GERD: gastroesophageal reflux disease.

^f^ND: neurodegenerative.

^g^WST: water swallowing test.

^h^EAT-10: Eating Assessment Tool.

^i^The number of participants reported in the paper was inconsistent.

^j^MWST: modified water swallowing test.

^k^RSWT: repetitive saliva swallowing test.

^l^ABI: acquired brain injury.

^m^FEES: fiberoptic endoscopic evaluation of swallowing.

^n^NG: neurogenic.

^o^NM: neuromuscular.

^p^TOR-BSST: Toronto Bedside Swallowing Screening Test.

^q^CVA: cerebrovascular accident.

### Modality

In our included studies, acoustic and vibratory signals were the primary modality sources used in dysphagia screening, as evidenced in 54% (13/24) and 38% (9/24) of the studies, respectively (Table 2). Additional sources included nasal airflow (2/24, 8%), electromyography (EMG; 2/24, 8%), strain and motion analysis (2/24, 8%), and optical method (1/24, 4%). Notably, 25% (6/24) of the studies used multimodal approaches, whereas the remaining 75% (18/24) focused on a single modality. The timeline trend shown in Figure 2 [35-58] indicates a consistent presence of acoustic modalities across various studies in general.

In our reviewed studies, various types of sound sensors (or microphones) were used to measure sound vibrations, including contact microphones [38,46,54,55], headset microphones [37,49], voice recorders from smart devices [36,43,52], accelerometers [35,44], and piezoelectric transducers [42]. Although there were variations in sensor placement across the studies, the suprasternal notch, thyroid cartilage, and cricoid cartilage were common anatomical landmarks for sensor placement. Inoue et al [42] introduced a multimodal system that incorporated nasal airflow measurements with laryngeal motion and sound analysis. In addition to a nasal cannula flow sensor, the system used a custom-designed piezoelectric sensor attached to the thyroid cartilage to detect throat motion and sound. Studies using accelerometers as acoustic transducers (2/24, 8%) typically focused on tracheal breathing sounds, positioning sensors at the suprasternal notch [35,44] and the intercostal space [44]. In contrast, studies using contact microphones (4/24, 17%) often emphasized vocal and swallowing sounds, placing sensors just below the level of cricoid cartilage [38] and at the level of thyroid cartilages [54,55]. Different algorithms were also introduced for signal segmentation, such as the discrete wavelet transform [55], the waveform dimension algorithm [44], and the hidden Markov model [35].

Accelerometers (8/24, 33%) were the most commonly used sensors in this review. Both dual-axial [45,47,56] and triaxial [38,39,50] accelerometers were used, aligned along the anterior-posterior and superior-inferior directions, with triaxial accelerometers additionally measuring the medial-lateral direction. Similar to the acoustic sensors, these sensors were typically placed at the level of the cricoid cartilage and just below the thyroid cartilage. Signal preprocessing primarily involved bandpass filtering and amplification in addition to denoising techniques such as finite impulse response filters [39] and mother wavelet transforms [50]. Signal segmentation or clipping was conducted either manually or automatically. In addition, some studies (2/24, 8%) developed custom biomotion or biophysical sensors. Ramírez et al [48] designed a flexible strain sensor using piezoresistive material composed of palladium nanoislands on single-layer graphene, which was applied to the submental region below the chin. On the other hand, Inoue et al [42] used a custom-made piezoelectric bending sensor to measure laryngeal motion that was positioned on the thyroid cartilage. However, the authors did not provide details on the sensors.

**Table 2:** Instrument modalities and screening protocols of the included studies.

Study	Source	Instrument	Task type	Protocol
Aboofazeli and Moussavi [35]	Acoustics	Accelerometer (measured trachea sound)	Eating or drinking Others	Eating or drinking: 5-mL boluses of semisolid, thick liquid, and thin liquid Others: breathing sound was recorded
Basiri et al [36]	Acoustics	Voice recorder	Swallowing Others	Swallowing: —^a^ Others: breathing
Cesarini et al [37]	Acoustics	Headset microphone	Speaking	Pronouncing the vowels “/a/” and “/e/” until breath shortening Reading 3 sentences in Italian with different consonant preponderance
Donohue et al [38]	Acoustic+vibratory	Contact microphone+triaxial accelerometer	Drinking	3-mL thin liquid boluses Self-selected “comfortable” cup sips of thin liquid
Dudik et al [39]	Vibratory	Triaxial accelerometer	Drinking	Water, nectar-thick liquid, and honey-thick liquid Head in neutral and chin-tuck position
He et al [40]	Vibratory	Vibration transducer used in a bone conduction headset	Speaking	Pronouncing 3 vowels Reading a simple text Reading a complicated tongue twister
He et al [41]	Acoustics	Vibration transducer	Speaking	Pronouncing the vowels “/a/,” “/e/,” and “/o/”
Inoue et al [42]	Nasal airflow+laryngeal motion+acoustics	Nasal cannula pressure sensor+piezoelectric sensor (to record both motion and sound)	Eating or drinking	Several types of test food and water
Kim et al [43]	Acoustics	Microphone of iPad (voice recorder)	Speaking Others	Speaking: sustained vowel “/e/” for 3 s, pitch elevation with “/eee/” from a low to a high pitch, and counting from 1 to 5 Others: voluntary coughing with maximal effort
Lazareck and Moussavi [44]	Acoustics+nasal airflow	Accelerometer+nasal cannula pressure sensor	Eating or drinking	5-10 spoons of 5-mL semisolid Single-bolus-sized sip of thick and thin liquid
Lee et al [45]	Vibratory+nasal airflow	Dual-axial accelerometer+nasal cannula pressure sensor	Eating or drinking	Thin liquid, nectar, spoon-thick liquid, and solid
Miyagi et al [46]	Acoustics	Contact microphone	Drinking	3 mL of water
Nikjoo et al [47]	Vibratory	Dual-axial accelerometer	Drinking	2-3 teaspoons of thin liquid barium Neutral head position
Ramírez et al [48]	Strain+myoelectric	Piezoresistive+sEMG^b^	Eating or drinking	10 mL of water, 15 mL of yogurt, and 6 g of crackers
Roldan-Vasco et al [49]	Acoustics	Headset microphone	Speaking	Sustaining vowels “/a/,” “/e/,” “/i/,” “/o/,” and “/u/” for at least 3 s Rapid repetition of the syllables “pa-ta-ka” Spontaneous monologue of approximately 90 s
Roldan-Vasco et al [50]	Vibratory+myoelectric	Triaxial accelerometer+sEMG	Eating or drinking	5, 10, and 20 mL of water and yogurt
Roldan-Vasco et al [51]	Myoelectric	sEMG	Eating or drinking	5, 10, and 20 mL of yogurt, water, and saliva and 3 g of cracker
Saab et al [52]	Acoustics	Microphone of iPhone (voice recorder)	Speaking	Performing speech components of the NIHSS^c^, including tests of articulation, naming, repetition, and comprehension Sustaining vowels “/a/,” “/e/,” “/i/,” “/o/,” and “/u/” for at least 3 s
Sakai et al [53]	Optical	Camera (photo) of iPad	Others	Sitting upright with chin in neutral position
Spadotto et al [54]	Acoustics	Contact (electret) microphone	Drinking	10 mL of water
Spadotto et al [55]	Acoustics	Contact microphone	Drinking	10 mL of water
Steele et al [56]	Vibratory	Dual-axial accelerometer	Drinking	6 sips of water 4 oz of thin liquid barium 3 sips of 4 oz of mildly thick, a teaspoon of moderately thick, and a teaspoon of extremely thick barium
Wang et al [57]	Vibratory	Vibration transducer of bone conduction headset	Speaking	—
Zhao et al [58]	Vibratory	Vibration transducer of bone conduction headset	Speaking	Pronouncing the vowel “/a/” for as long as possible

^a^Information not available.

^b^sEMG: surface electromyography.

^c^NIHSS: National Institutes of Health Stroke Scale.

Nasal airflow measurement and EMG were additional modalities used to identify dysphagia, both of which were featured in 8% (2/24) of the studies. Nasal airflow monitoring was facilitated by a nasal cannula connected to a pressure transducer [42,44,45]. This method provided insights into the respiratory-swallowing coordination, laryngeal closure timing, risk of aspiration, and dysphagic compensatory mechanisms such as prolonged swallow apnea [60,61]. Conversely, EMG studies primarily focused on the infrahyoid muscle [50,51] and suprahyoid muscle groups [48,50,51], including the mylohyoid and geniohyoid muscles. The suprahyoid muscles initiate swallowing and protect the airway during the process, whereas the infrahyoid muscles stabilize the hyoid bone and assist in lowering the larynx after swallowing [62]. They play an important role in the positioning of the hyoid bone, swallowing, and speech.

### Protocol

To identify dysphagia, the screening protocols can be broadly categorized into swallowing and nonswallowing tasks. Swallowing tasks, which appeared in 62% (15/24) of the studies, involved activities such as eating, drinking, and saliva swallowing. Nonswallowing tasks, featured in 46% (11/24) of the studies, included speaking and other maneuvers, such as coughing or simply breathing.

The swallowing tasks exhibited considerable variation in protocols, particularly regarding the volume and consistency (viscosity) of the food or liquid to be swallowed. Some studies (3/24, 13%) used relatively simple protocols in which participants were instructed to swallow 10 mL of water [54,55] or thin liquid barium [47]. Donohue et al [38] asked the participants to swallow 3 mL of thin liquid and then self-selecting the volume of the sips they would swallow of that liquid. Several studies (4/24, 17%) evaluated the influence of different liquid thicknesses. Steele et al [56] tested 6 sips of water followed by 6 sips of thin liquid barium and 3 sips each of mildly thick, moderately thick, and extremely thick barium. Similarly, Dudik et al [39] presented participants with water, nectar-thick liquid, and honey-thick liquid. Some protocols incorporated a spectrum of consistencies from liquid to solid. The studies conducted by Aboofazeli and Moussavi [35] and Lazareck and Moussavi [44] asked participants to swallow a 5-mL semisolid bolus and sips of thick and thin liquid. Lee et al [45] progressively fed participants thin, nectar-thick, and spoon-thick liquids followed by solids. Other studies involved saliva swallowing, different volumes of water, yogurt, and crackers in their protocols [48,50,51]. Notably, Dudik et al [39] compared the neutral head and chin-tuck positions during the swallowing tasks.

Pronouncing vowels is the most common nonswallowing task, which appeared in 29% (7/24) of the studies [37,40,41,43,49,52,58] despite some variations. Participants were typically asked to articulate the vowels “/a/,” “/e/,” “/i/,” “/o/,” and “/u/” (in phonemic alphabets: “/eɪ/,” “/iː/,” “/aɪ/,“ “/oʊ/,” and “/u:/”). Some studies (3/7, 33%) required participants to pronounce only 2 or 3 of these vowels. The protocols varied, with 33% (2/6) of the studies simply asking participants to articulate the vowels [40,41], whereas the others (4/6, 67%) required them to speak at different pitch levels (high-pitch gliding) [43], sustain the vowel sound for 3 seconds [43,52], or pronounce the vowel for as long as possible [58] or until they needed to take a breath [37]. In addition, participants were tested on consonants [37] and syllables [49]. More complex reading tasks were also introduced, including text reading [40], counting (1 to 5) [43], tongue twisters [40], and reading a monologue for 90 seconds [49]. Furthermore, Saab et al [52] administered the speech components of the National Institutes of Health Stroke Scale [63], which included tests of articulation, naming, repetition, and comprehension.

### Model

In synthesizing the review of the models, we can categorize those used in the included studies into deep learning (3/24, 12%) and traditional machine learning models (21/24, 88%). Dudik et al [39] created a complex neural network that combines 2 directions of accelerometric data. This network has multiple layers that process the data in different ways before making a final classification. On the other hand, some studies (2/24, 8%) converted the collected signals into “images,” which were then processed using convolutional neural networks. Saab et al [52] converted signals into red-green-blue mel-spectrogram images, with color indicating the spectral (intensity of the frequency component) magnitude of the time-frequency domain (image space) and 3-channel mel-spectrogram images involving the depth-wise concatenation of 3 monochrome mel-spectrograms. These were input into an ensemble network of the DenseNet121 and ConvNeXtTiny models to identify dysphagia. Similarly, Kim et al [43] converted acoustic signals to short-time Fourier transform and mel-frequency cepstral coefficient (MFCC) spectrograms via fast Fourier transform, mel filter bank, and inverse fast Fourier transform. The MFCC was derived by applying a discrete cosine transform to the log mel-spectrogram. The researchers then input the short-time Fourier transform and MFCC into a convolution-batch normalization–rectified linear unit block and DenseNet121 block, respectively, merging them using concatenation followed by fully connected layers. They created models for each task, evaluated them separately, and ensembled the models using a soft voting method.

Feature extraction is an important step in traditional machine learning. The included studies highlighted a diverse range of predetermined features to characterize signals, particularly in acoustics and accelerometry. In the time domain, commonly extracted features such as SD, variance, root mean square, waveform length, and zero-crossings could provide insights into the amplitude and behavior of the signal. Frequency domain features such as peak, mean, and median frequency, as well as total energy, were extracted for spectral representation of signals. Spectrograms and wavelet analyses in the time-frequency domain could capture both temporal and spectral variations of the signals. Some studies (2/24, 8%) considered information-theoretical domain features, which included dispersion ratio, normality, Lempel-Ziv complexity, and entropy [38,47]. For acoustics and airflow information, domain-specific features were also used. Audio (acoustic) domain features included jitter, shimmer, pitch, amplitude, and pitch perturbation quotients [37,49,57,58], whereas airflow features encompassed maximum hyolaryngeal excursion and air volume [45]. After collecting data, researchers need to choose which aspects of the data (ie, features) are most important. This process, known as feature selection or reduction, helps manage the large amount of information collected [64]. Principal-component analysis is one of the famous methods for feature selection to retain most of the original variance [36,50,57,58]. Some studies (4/24, 17%) used statistical methods such as *t* tests or regressions to evaluate the feature importance in relation to the target variable [40,44,49,53]. These statistical methods help identify the most relevant feature and reduce computational demand. There were other feature selection methods used, such as the minimum redundancy, maximum relevancy method [50,51] and precise matching analysis [40]. Other feature domains and feature extraction and selection methods are detailed in Multimedia Appendix 4 [35-58].

Support vector machine (SVM) was used in 62% (15/24) of the studies as the traditional machine learning model, although different kernel functions were applied across these studies. SVM handles nonlinear data by using the kernel function to identify an optimal hyperplane in the high-dimensional space for classification. The radial basis function kernel was used on the SVM model in 25% (6/24) of the studies [37,42,46,49,54,58], whereas Roldan-Vasco et al [49] conducted a comparison of the radial basis function with linear and sigmoid kernels. In addition, Nikjoo et al [47] constructed SVMs on each feature domain and ensembled the models using a reputational classification approach. Linear discriminant analysis (LDA), which was featured in 21% (5/24) of the studies [44,45,49,56], shares similarities to SVM as both methods construct a decision boundary to classify the feature space. However, unlike SVM, which maximizes the margin between classes, LDA seeks to maximize the ratio of between-class variance to within-class variance. In one study, Lazareck and Moussavi [44] conducted LDA using 11 features extracted from the time-frequency domain of signals, which were segmented based on the waveform dimension trajectory. In another study, Lee et al [45] trained and calibrated the LDA model using variant measures of Euclidean and Mahalanobis distance.

Statistical models such as logistic regression and Bayesian methods offer probabilistic output and better interpretability, in addition to lower computational cost. For instance, Spadotto et al [55] conducted a time-frequency analysis based on the discrete wavelet transform and classified the signal using the Bayesian method. On the other hand, Donohue et al [38] extracted 22 features from the time, frequency, time-frequency, and information-theoretical domains after principal-component analysis and evaluated the classification performance of naïve Bayes and logistic regression comparing them with SVM and decision tree. Tree-based models, including decision tree, random forest, and boosting models such as Adaptive Boosting (AdaBoost) and Extreme Gradient Boosting (XGBoost), are potent and interpretable models that perform classification tasks using hierarchical structures of conditional control algorithms. In this review, Roldan-Vasco et al [49] assessed the performance of decision tree and random forest on the features extracted from the audio, articulation, diadochokinetic, and prosody domains. Boosting models, on the other hand, combine weaker learners (or trees) to form stronger learners. Both AdaBoost and XGBoost were featured in our reviewed studies [48,50,51,57], demonstrating their effectiveness in classifying dysphagia. Neural networks, which are computer systems modeled after the human brain, can learn complex patterns. However, they require large amounts of data and significant computing power to function effectively. Examples of such networks used in this review included multilayer perceptron, artificial neural network, and probabilistic neural network [45,49,58].

### Performance

**Figure 5:**
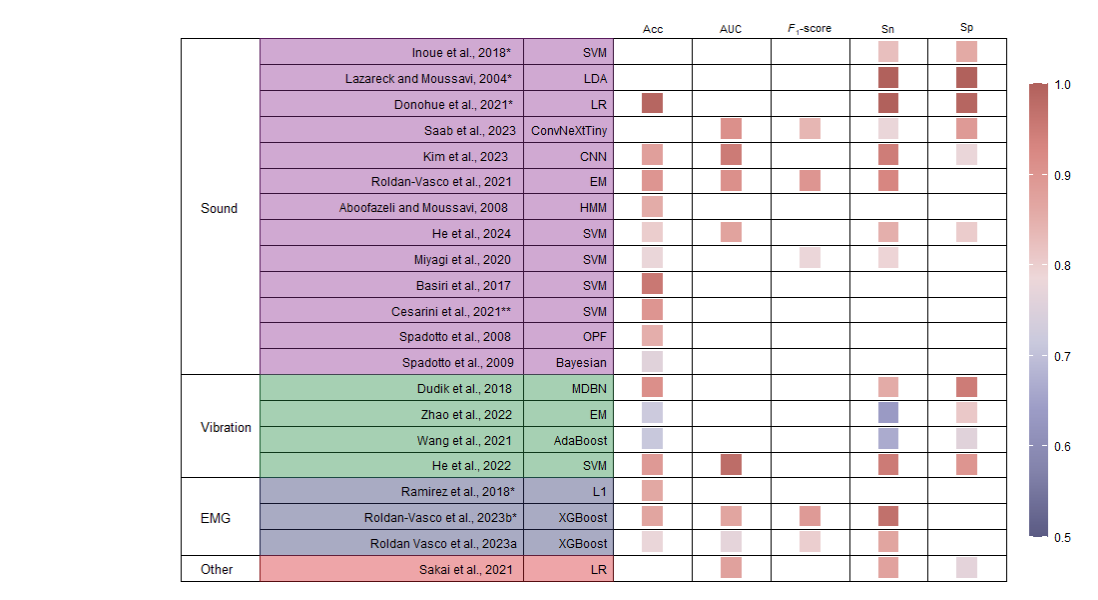
Heat map comparing the classification performance between models in the included studies [35-44,46,48-55,57,58]. *The study involved multimodality. **The study reported that all models reached an accuracy of >90% but did not provide their exact values. Acc: accuracy; AdaBoost: Adaptive Boosting; AUC: area under the receiver operating characteristic curve; CNN: convolutional neural network; EM: ensemble model; EMG: electromyography; HMM: hidden Markov model; L1: Lasso regularization; LDA: linear discriminant analysis; LR: logistic regression; MDBN: multilayer deep belief network; OPF: optimum-path forest; Sn: sensitivity; Sp: specificity; SVM: support vector machine; XGBoost: Extreme Gradient Boosting.

Figure 5 [35-44,46,48-55,57,58] (more detailed information is available in Multimedia Appendix 5 [35-58]) shows the performance of a model that was evaluated on an individual basis and was deemed the best-performing model in the study. This figure includes a list of 88% (21/24) of the studies along with specific settings such as selected features and tasks. Most of the studies reported the accuracy metric (17/21, 81%), whereas fewer reported the *F*_1_-score and AUC. Both accuracy and *F*_1_-score represent the proportion of correctly classified observations. However, the *F*_1_-score, which is the harmonic mean of precision and recall, is more robust to class imbalance even though it is more challenging to interpret. On the other hand, the AUC measures the performance of a binary classifier at different thresholds and serves as a metric to represent the model’s discriminative power or separability. Sensitivity and specificity were reported in 71% (15/21) and 52% (11/21) of the studies, respectively. While both sensitivity and specificity are crucial parameters in the evaluation of diagnostic or screening tests, their relative importance can vary depending on the context of the application. In the context of dysphagia screening, we attributed a higher degree of importance to sensitivity. When an individual tests positive in the screening, they may undergo additional tests for confirmation. However, missing a diagnosis could lead to higher costs, both financially and in terms of patient health outcomes.

The models in the included studies generally exhibited high performance. Among the multimodal studies, Donohue et al [38] demonstrated exceptional performance, with a logistic regression model achieving an accuracy of 0.99 when integrating sound and vibration data. Similarly, Inoue et al [42] explored the combination of nasal airflow, biomotion, and sound, achieving a sensitivity of 0.824 and specificity of 0.86, In the context of multimodal studies involving EMG, Roldan-Vasco et al [49] achieved an accuracy of 0.90 using an ensemble model that incorporated various features across all tasks. In addition, Roldan-Vasco et al [50] demonstrated the efficacy of XGBoost in analyzing vibration and EMG data, achieving an accuracy of 0.87. Regarding unimodal sound studies, Basiri et al [36] reported an excellent accuracy of 0.9565 using SVM, whereas Aboofazeli and Moussavi [35] achieved an accuracy of 0.855 using the hidden Markov model.

The models using vibration as a modality exhibited a range of classification accuracy, from 0.712 to 0.913. Dudik et al [39] used a multilayer deep belief network and achieved an accuracy of 0.913 with a sensitivity of 0.949. He et al [40] used SVM, reporting an accuracy of 0.892 and an AUC of 0.977. In contrast, 8% (2/24) of the studies achieved a lower accuracy of 0.721 [58] and 0.712 [57] using the ensemble model and AdaBoost, respectively.

Multimodal studies demonstrated superior performance compared to unimodal studies. Specifically, multimodal studies incorporating sound achieved an accuracy of 0.99, which is higher than that of unimodal studies focusing solely on sound (0.88-0.90). A similar trend was observed for EMG. Studies that combined EMG with other modalities attained an accuracy between 0.86 and 0.87, whereas EMG alone achieved an accuracy of 0.78. Inoue et al [42] compared their own multimodal results with their unimodal findings. The multimodal performance was comparable to that of acoustic measurements but notably superior to that of airflow and biomotion measurements in isolation. Furthermore, Roldan-Vasco et al [50] demonstrated that integrating accelerometry and surface EMG improved accuracy by 5% to 21% compared to using either method independently.

It should be noted that 12% (3/24) of the studies [45,47,56], which solely classified per episodes (ie, safe vs unsafe swallows), were not included in Figure 5 [35-44,46,48-55,57,58]. Nikjoo et al [47] achieved a promising accuracy of 80.48% and a sensitivity of 97.1%, with a moderate specificity of 64%. By using the Mahalanobis linear discriminant classifier, which was the best model, Lee et al [45] achieved an accuracy of 84.2% when the bolus clearance of the pyriform sinus scale was used as the ground truth. Steele et al [56] focused on impaired thin liquid swallowing safety, achieving an identification sensitivity of 90.4% and a specificity of 60.0%. In total, 8% (2/24) of the studies [57,58], which used throat vibration sensors, demonstrated relatively poor performance, as illustrated in Figure 5 [35-44,46,48-55,57,58]. The authors attributed this suboptimal performance to the high individual variation in vibration-based speech features, potentially influenced by factors such as age.

## Discussion

### Principal Findings

**Figure 6:**
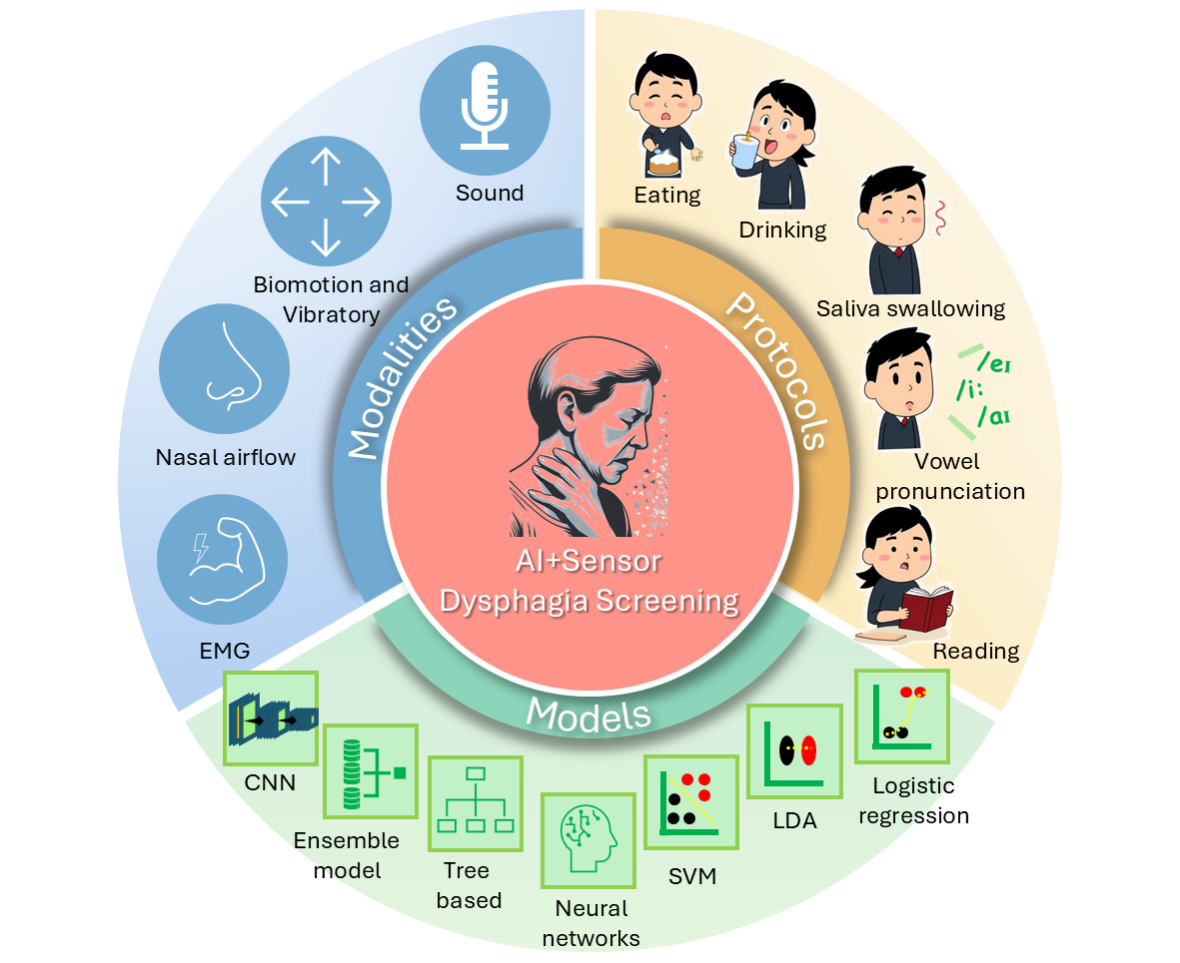
A fan chart illustrating the context and concepts of the 3 key domains of models, modalities, and protocols. AI: artificial intelligence; CNN: convolutional neural network; EMG: electromyography; LDA: linear discriminant analysis; SVM: support vector machine.

This systematic scoping review analyzed 24 studies on AI and sensor-based dysphagia screening, illustrating important context and concepts regarding modalities, protocols, and models (Figure 6). Acoustic-based modalities were the most prevalent, with various instruments, such as contact microphones, headset microphones, voice recorders, and accelerometers, being used. In addition, EMG appears to be gaining recognition in recent studies. SVM emerged as the most frequently used AI model, although different kernel functions were used across the studies. Multimodal systems that used multiple types of data appeared to be superior to unimodal systems. Performance metrics varied widely, with accuracy ranging from 71.2% to 99%, AUC ranging from 0.77 to 0.977, and sensitivity ranging from 63.6% to 100%. However, it is crucial to note that the validity of these metrics may be compromised in some studies due to the small testing sample size, particularly for the dysphagia group. Most studies (17/24, 71%) had <60 participants in the dysphagia group. While no clear performance trends were observed between traditional machine learning and deep learning approaches, more recent publications showed a tendency toward using deep learning and ensemble models. The primary focus of this review was on the classification of individuals as having dysphagia or not. Some studies (3/24, 13%) used a 2-step approach, initially classifying swallows as safe or unsafe before making a final determination regarding dysphagia. This 2-step approach could be advantageous as individuals with dysphagia might not always produce “unsafe” swallowing episodes. Testing various swallowing tasks provides additional evidence to support the screening results. On the other hand, our methodological quality assessment raised several concerns regarding the studies reviewed. These concerns included inadequate declaration of sampling approaches, lack of blinding in class label assignment, insufficient hyperparameter tuning and handling of class imbalances, and limited external testing or validation. These methodological issues highlight the need for more transparent reporting and to enhance the reliability and generalizability of the research in future studies.

### Implications and Perspectives

While our review primarily focused on geriatric dysphagia, it is crucial to consider the unique challenges faced by specific subpopulations, particularly patients with dementia and children. For patients dementia, the progressive decline in cognitive function can affect their ability to understand and respond to questionnaires or follow complex instructions during clinical examinations. Currently, noninstrumental clinical observation and questionnaires remain the primary screening methods for this population. The modified Mann Assessment of Swallowing Ability was designed to assess dysphagia in patients with mild to moderate dementia [65]. However, it might fail to account for individuals with severe cognitive impairment, and its performance varies across different levels of cognitive decline [66].

Dysphagia is a complex disorder with diverse manifestations originating from the oral, pharyngeal, and esophageal regions, as well as neurological, coordination, structural, and sarcopenic factors [67]. This complexity necessitates a thoughtful selection of screening modalities as the right combination can not only improve overall screening accuracy but also help pinpoint the specific anatomical regions affected and the underlying nature of the swallowing difficulty. For oral and pharyngeal regions, cervical auscultation or acoustic-based techniques are more common. These techniques detect abnormal sound or vibration patterns linked to alteration in laryngeal vestibule closure and opening and hyoid bone movement [68,69]. Temporal analysis of these swallowing sounds or vibratory biomotion signals could identify neurological and coordination factors of dysphagia. To assess structural abnormalities in the oral, pharyngeal, and esophageal regions, ultrasound imaging offers real-time visualization and is particularly useful for identifying anatomical changes that may contribute to swallowing difficulties [70]. For suspected sarcopenia, which could affect the swallowing mechanism, optical or camera-based systems can assess muscle mass and morphological changes in different stages of swallowing [53]. In addition, EMG can offer valuable insights into muscle weakness, activation patterns, and coordination issues [71]. It also has the potential to be further developed into a biofeedback tool for dysphagia rehabilitation [72]. Considering the complex nature of dysphagia, a multimodal approach that integrates various sensing technologies can effectively capture the full range of swallowing abnormalities across different regions and underlying causes. By leveraging AI, this approach can be further enhanced through the integration of diverse data types, extraction of relevant features from each modality, and temporal and spatial analysis of swallowing patterns, ultimately providing personalized risk assessments.

The integration of AI and multimodal sensor-based technologies in dysphagia screening has the potential to revolutionize clinical practice, offering numerous benefits for both patients and health care providers [26,73]. First, these advanced screening methods can enhance the detection of subtle abnormalities, providing objective and consistent assessments, which may ultimately lead to improved accuracy and early intervention. Second, an AI-based sensor system could automate screening and triage processes, significantly alleviating the workload of health care professionals. Third, the incorporation of these technologies in the form of wearables could enable continuous monitoring. This allows for the capture of intermittent or subtle difficulties that might be overlooked in a one-off traditional assessment given that dysphagia can be a dynamic condition that presents differently in the same individual under different circumstances. Fourth, this functionality could pave the way for more personalized treatment plans and facilitate remote monitoring, which is particularly beneficial for patients in underserved areas or during a pandemic. The potential for tele-swallowing assessments [74] and home-based screening not only improves accessibility but also enhances patient comfort by reducing the need for travel and minimizing the anxiety associated with traditional invasive procedures such as the VFSS and FEES [75].

From a health care cost perspective, the implementation of AI and sensor-based screening methods could reduce the reliance on costly diagnostic procedures, and early identification could potentially prevent complications such as aspiration pneumonia, thereby decreasing hospital admissions and enhancing patient outcomes. Furthermore, the reduced need for specialists such as speech or occupational therapists for bedside screenings or tests could contribute to cost-effectiveness. The integration of electronic health record systems presents additional opportunities for these technologies given that dysphagia can be associated with different underlying factors and comorbidities such as malnutrition, intubation, stroke, brain injury, dementia, and sarcopenia [76,77], requiring a holistic management and treatment plan [67]. Future studies might leverage language models to facilitate automated clinical reports and adaptive treatment planning [78-81].

### Hurdles and Opportunities

Despite the promising potential for AI-based dysphagia screening, several technological challenges have hindered the pathway toward the anticipated impact. Key challenges include issues of generalizability, robustness, limitations in sample size, biases in model training, variations in screening protocols, and the need for real-time processing capabilities. Our review revealed an absence of external tests across all studies. While many models demonstrated good performance in internal tests, their ability to maintain accuracy across different screening environments, protocols, or patient populations and, thus, their generalizability or external validity remain uncertain, posing challenges to real-world applicability. Sample size, for both training and testing, presents another set of challenges. Small datasets can lead to overfitting and induced bias in the performance evaluation. Wang et al [82] suggested that a test sample size of 98 might be necessary for deep learning, as estimated based on statistical heuristics. Data augmentation and other techniques such as undersampling, penalization, and Monte Carlo simulation could be used to address class imbalance problem because of scarcity of patients with dysphagia in the dataset. The fact that some patients with dysphagia cannot perform certain swallowing tasks or maintain certain postures, resulting in missing data, could contribute to model bias. For instance, one study regulated the consistencies and volume of the food to be swallowed to maintain a safe level for the patient group [50], whereas others had their protocols controlled by the attending clinicians [45,47]. The model may tend to inadvertently learn to correlate the lack of specific data with the classification of an individual as a patient rather than identifying pertinent features from the data that have been collected [83]. A standardized screening protocol is indeed essential. There have been protocols proposed for this application [84], and some studies have referenced various relevant protocols such as the International Dysphagia Diet Standardisation Initiative [85] and National Institutes of Health Stroke Scale [63]. Nonetheless, additional research is required to understand the relationship among intake consistency; intake volume; and their impact on swallowing function, biomechanics, and physiology [86,87].

Efforts should be directed toward creating large, more diverse datasets of dysphagia presentations. Prioritizing external validation studies is also crucial to assess the generalizability and robustness of AI models across diverse clinical settings and patient populations [88]. Moreover, all studies in this review focused on a single swallowing assessment. However, a long-term continuous assessment could be more beneficial as geriatric dysphagia is a gradual deterioration process. The need for real-time processing capabilities presents a significant hurdle in translating AI-based dysphagia screening from research to clinical practice. The integration of AI models into Internet of Things systems, the implementation of edge computing, the development of lightweight models suitable for real-time analysis, and usability tests in clinical settings are crucial steps [89,90] that have yet to be fully addressed. Tsujimoto et al [91] explored the use of a smartphone-based, neck-worn monitoring device for swallowing activities (NeW–Monitoring Device for Swallowing Activities, GOKURI neckband; PLIMES Inc) to monitor the swallowing frequency of food of different consistencies in daily life and demonstrated its feasibility in continuous monitoring. In addition, the advent of soft and flexible materials in sensor technology could significantly improve comfort and compliance, while also reducing noise and motion artifacts. These advances pave the way for more extensive long-term analysis [92-94], whereas noncontact methods such as optical and depth cameras might have similar advantages [28,29]. However, a critical challenge remains in ensuring both consistent sensor placement and proper prestretching of the device, particularly for devices adhered to the neck region. Addressing this issue is crucial to enable reliable repeated measurements to track disease progression or treatment efficacy. Broader challenges include issues such as data privacy, user compliance, maintenance, user-friendliness, and technological resistance. Wearable technologies and other assistive technologies have faced compliance issues among older adults, especially those with dementia and agitation [95,96]. Designing user-friendly interfaces and incorporating persuasive features for older adults could enhance usability and adherence [97].

Future research should explore innovative AI architectures and advanced sensing technologies, with a particular focus on multimodal approaches that integrate data from diverse sensors to enhance accuracy and robustness [98]. The clinical utility of the system could be further improved through model distillation [99] against electronic health records, demographic information, psychographic data, and environmental factors [100,101]. This comprehensive approach could lead to more precise assessments, advancing the field toward precision telemedicine. Apart from screening and monitoring swallowing function, these sensor technologies have potential applications as biofeedback-based controllers. Such applications could also enable novel approaches to swallowing rehabilitation through various modalities of serious games, such as biofeedback-based video and virtual reality games [102-104].

### Comparison to Prior Reviews

This review extends the existing body of literature. While previous reviews have focused on specific aspects, our study provides a comprehensive overview of the current state of the research in dysphagia classification using AI and instruments. Lai et al [27] conducted a meta-analysis on the diagnostic accuracy of wearable technology for identifying aspiration risk exacerbated by dysphagia, whereas So et al [26] reviewed acoustics and accelerometric instruments for classifying swallow and nonswallow tasks. Li et al [68] conducted a narrative review of the acoustic theory foundation and applications for monitoring swallowing sound. We found additional modalities in our review, such as nasal airflow, EMG, and biomotion measured using piezoresistive sensors, providing a broader perspective. Another narrative review by Wu et al [73] provided a comprehensive overview on different noninvasive sensors for swallowing assessments. Consistent with previous reviews, we found that SVM was the most common model used across the studies. The variations in screening protocols, the limitation of small sample sizes, and the lack of external tests were also common problems. On the other hand, Rafeedi et al [93] reviewed proof-of-concept studies from an engineering perspective, exploring the application of soft sensors (referred to as “epidermal sensors”) for potential long-term swallowing monitoring.

### Limitations of This Review

This review has several limitations. First, our search strategy was restricted to papers in English, potentially excluding relevant studies published in other languages. We also excluded certain types of publications, such as conference abstracts, commentaries, perspectives, and book chapters, which may have contained relevant information. The scope of our review was further limited by the exclusion of studies focusing on pediatric or infant dysphagia, as well as postextubation-induced dysphagia. While necessary to maintain focus, it may have omitted important findings and applications in these specific populations. A significant challenge in this review was the heterogeneity among the studies, particularly in terms of signal processing, feature extraction, and feature selection methodologies. This heterogeneity made it difficult to synthesize and compare results across studies effectively. In addition, our focus on classification studies may have led to the exclusion of relevant studies that used AI models for other purposes, such as signal segmentation or severity quantification [91,105-108].

While the assessment of the quality of the included studies is a strength of our scoping review, the application of the QUADAS-2 has limitations as this tool might not be optimally designed for evaluating AI-based studies. The problem of reporting guidelines for medical AI research, underscored by Kolbinger et al [109], emphasizes the necessity for a more suitable quality assessment tool in this field. In this review, we addressed this issue by adapting the QUADAS-2 and incorporating a relevant *model* domain (ie, QUADAS-2+M) to provide additional information. However, this modification still necessitates further validation. In addition, the original QUADAS-2 and our proposed QUADAS-2+M framework aggregate multiple signaling questions into a single risk-of-bias grade for each domain. While this approach provides a comprehensive overview, it may obscure specific areas of concern. A more granular breakdown of signaling questions could offer valuable insights. Specifically, disaggregating the assessment of hyperparameter tuning, class imbalance, and missing data handling would provide a better understanding of potential biases in AI studies. In brief, the absence of sufficient hyperparameter tuning can lead to suboptimal model configurations, potentially failing to balance the bias-variance trade-off [110]. This may result in models that either fail to capture important patterns in the data or overfit to noise and irrelevant features, leading to biased predictions. Class imbalance, if not adequately addressed, can introduce significant bias as models tend to focus more on the majority class, potentially overlooking important patterns in the minority class [111]. This is particularly crucial in medical contexts where the minority class often represents the condition of interest [111]. Furthermore, failing to address missing data appropriately can introduce several significant biases, including selection bias and reduced statistical power [112]. These biases can collectively distort effect estimates and lead to invalid conclusions, potentially impacting the clinical applicability of AI models in dysphagia screening.

Moreover, we encountered challenges in data extraction from some materials science–oriented studies that focused primarily on sensor fabrication and characterization, with limited details on participant testing protocols or AI models. In some cases, critical information was moved to the supplementary materials or left out entirely, making comprehensive analysis challenging.

### Conclusions

This systematic scoping review highlights the emerging potential of AI and sensor-based technologies in dysphagia screening. The reviewed studies demonstrate promising advancements in developing more accessible, objective, and reliable screening tools that address some limitations of traditional methods. Key findings include the following:

1. A diverse range of modalities were used, with microphones and accelerometers being the most common sensors used.
2. Most studies (18/24, 75%) focused on per-individual classification rather than swallow event classification. A 2-step approach, from per swallow to per individual, might further improve screening accuracy.
3. Classic machine learning models, particularly SVM, were frequently used, whereas deep learning approaches have been gaining traction. Multimodal systems appeared to perform better than unimodal systems.
4. Performance metrics varied widely across the studies, with some reporting high accuracy and AUC values but often lacking comprehensive evaluation across all relevant metrics. It is also worth noting that the validity of evaluations for studies with a very small testing sample may be limited.

Several challenges remain. The methodological quality assessment revealed a high risk of bias in many studies, particularly in patient selection, blinding procedures, and model development. In addition, many studies did not test their AI systems in different settings or with different populations (ie, external validation and domain adaptation testing), which raises concerns about the transferability and real-world applicability of these AI-based systems. Future research should focus on improving methodological rigor (eg, sampling and blinding), addressing class imbalance issues, and conducting robust external validation studies. These technologies have potential to significantly enhance early detection and management of dysphagia, particularly in resource-constrained settings such as residential care homes.

## Appendix

Multimedia Appendix 1 PRISMA-ScR checklist.

Multimedia Appendix 2 Full search terms and queries for the systematic database search.

Multimedia Appendix 3 Search queries, hits, and entries from various databases.

Multimedia Appendix 4 Feature extraction, artificial intelligence model, and model training settings in the included studies.

Multimedia Appendix 5 Model performance.

## Abbreviations

AdaBoost: Adaptive Boosting
AI: artificial intelligence
AUC: area under the receiver operating characteristic curve
EMG: electromyography
FEES: fiberoptic endoscopic evaluation of swallowing
LDA: linear discriminant analysis
MFCC: mel-frequency cepstral coefficient
PRISMA-ScR: Preferred Reporting Items for Systematic Reviews and Meta-Analyses extension for Scoping Reviews
QUADAS-2: Quality Assessment of Diagnostic Accuracy Studies–2
QUADAS-2+M: modified version of the Quality Assessment of Diagnostic Accuracy Studies–2 including a fifth domain (model)
SVM: support vector machine
TRIPOD+AI: Transparent Reporting of a Multivariate Prediction Model for Individual Prognosis or Diagnosis + Artificial Intelligence
VFSS: videofluoroscopic swallowing study
XGBoost: Extreme Gradient Boosting
