# Supplementary material for: Current Technological Advances in Dysphagia Screening: Systematic Scoping Review
Source: J Med Internet Res. 2025 May 5;27:e65551. doi: 10.2196/65551 (PMC12089864; doi:10.2196/65551)
Supplement: Multimedia Appendix 2 [file jmir_v27i1e65551_app2.docx]

Search Terms

|  | Domain | Terms |
| --- | --- | --- |
| #1 | Participants | Dysphagia OR dysphagic OR "swallowing disorder*" OR "deglutition disorder*" OR “unsafe swallow*” |
| #2 | Model | “deep learning*” OR “machine learning*” OR “neural network*” OR transformer* OR detect* OR monitor* OR classif* OR recogni* OR discriminat* OR distinguish* |
| #3 | Instrument | sensor* OR sensing OR biosens* OR instrument* OR speech OR biomarker* OR voice OR acoustic* OR sound* OR microphone* OR “cervical auscultation” OR pressure OR stress OR strain OR stretch* OR accelero* OR vibrat* OR mechanomyo* OR kinematic* OR displacement* OR camera OR optical OR imaging OR image* OR video OR ultraso* OR sonograph* OR sonic OR EMG OR electromyograph* OR wearable OR airflow OR biosignal* |
| #4 | Outcomes | sensitivity OR specificity OR precision* OR recall OR accuracy OR "positive predictive" OR "negative predictive" OR PPV OR NPV OR AUC OR AUROC OR PR?AUC OR AUC?PR OR "area under curve" OR "receiver operating" OR "diagnostic odds ratio" OR DOR OR F1?score |
| #5 | Exclusion | paediatric* OR pediatric* OR infant OR birth OR childhood OR children OR “cerebral palsy” OR intubat* OR extubat* OR postextubation OR post-extubation |

(#1 AND #2 AND #3 AND #4) NOT #5
