# Supplementary material for: Current Technological Advances in Dysphagia Screening: Systematic Scoping Review
Source: J Med Internet Res. 2025 May 5;27:e65551. doi: 10.2196/65551 (PMC12089864; doi:10.2196/65551)
Supplement: Multimedia Appendix 3 [file jmir_v27i1e65551_app3.docx]

Search Date: 04 Jul 2024

Search Queries for PubMed

| **Number** | **Search Terms** | **Search Hits** |
| --- | --- | --- |
| #1 | "Dysphagia"[Title/Abstract] OR "dysphagic"[Title/Abstract] OR "swallowing disorder*"[Title/Abstract] OR "deglutition disorder*"[Title/Abstract] OR "unsafe swallow*"[Title/Abstract] | 39,300 |
| #2 | "deep learning*"[Title/Abstract] OR "machine learning*"[Title/Abstract] OR "neural network*"[Title/Abstract] OR transformer*[Title/Abstract] OR detect*[Title/Abstract] OR monitor*[Title/Abstract] OR classif*[Title/Abstract] OR recogni*[Title/Abstract] OR discriminat*[Title/Abstract] OR distinguish*[Title/Abstract] | 5,804,172 |
| #3 | "sensor*"[Title/Abstract] OR "sensing"[Title/Abstract] OR "biosens*"[Title/Abstract] OR "instrument*"[Title/Abstract] OR "speech"[Title/Abstract] OR "biomarker*"[Title/Abstract] OR "voice"[Title/Abstract] OR "acoustic*"[Title/Abstract] OR "sound*"[Title/Abstract] OR "microphone*"[Title/Abstract] OR "cervical auscultation"[Title/Abstract] OR "pressure"[Title/Abstract] OR "stress"[Title/Abstract] OR "strain"[Title/Abstract] OR "stretch*"[Title/Abstract] OR "accelero*"[Title/Abstract] OR "vibrat*"[Title/Abstract] OR "mechanomyo*"[Title/Abstract] OR "kinematic*"[Title/Abstract] OR "displacement*"[Title/Abstract] OR "camera"[Title/Abstract] OR "optical"[Title/Abstract] OR "imaging"[Title/Abstract] OR "image*"[Title/Abstract] OR "video"[Title/Abstract] OR "ultraso*"[Title/Abstract] OR "sonograph*"[Title/Abstract] OR "sonic"[Title/Abstract] OR "EMG"[Title/Abstract] OR "electromyograph*"[Title/Abstract] OR "wearable"[Title/Abstract] OR "airflow"[Title/Abstract] OR "biosignal*"[Title/Abstract] | 6,388,225 |
| #4 | "sensitivity"[Title/Abstract] OR "specificity"[Title/Abstract] OR "precision*"[Title/Abstract] OR "recall"[Title/Abstract] OR "accuracy"[Title/Abstract] OR "positive predictive"[Title/Abstract] OR "negative predictive"[Title/Abstract] OR "PPV"[Title/Abstract] OR "NPV"[Title/Abstract] OR "AUC"[Title/Abstract] OR "AUROC"[Title/Abstract] OR "pr auc"[Title/Abstract] OR "auc pr"[Title/Abstract] OR "area under curve"[Title/Abstract] OR "receiver operating"[Title/Abstract] OR "diagnostic odds ratio"[Title/Abstract] OR "DOR"[Title/Abstract] OR "f1 score"[Title/Abstract] | 2,143,467 |
| #5 | "paediatric*"[Title/Abstract] OR "pediatric*"[Title/Abstract] OR "infant"[Title/Abstract] OR "childhood"[Title/Abstract] OR "children"[Title/Abstract] OR "cerebral palsy"[Title/Abstract] OR "intubat*"[Title/Abstract] OR "extubat*"[Title/Abstract] OR "postextubation"[Title/Abstract] OR "post-extubation"[Title/Abstract] | 1,990,540 |
| #6 | #1 AND #2 | 7,089 |
| #7 | #6 AND #3 | 2,749 |
| #8 | #7 AND #4 | 431 |
| #9 | #8 NOT #5 | 251 |
| #10 | #9 Filter: English | 248 |

Search Queries for Web of Science

| **Number** | **Search Terms** | **Search Hits** |
| --- | --- | --- |
| #1 | TS=(Dysphagia OR dysphagic OR "swallowing disorder*" OR "deglutition disorder*" OR “unsafe swallow*” ) | 38,545 |
| #2 | TS=(“deep learning*” OR “machine learning*” OR “neural network*” OR transformer* OR detect* OR monitor* OR classif* OR recogni* OR discriminat* OR distinguish* ) | 10,824,319 |
| #3 | TS=(sensor* OR sensing OR biosens* OR instrument* OR speech OR biomarker* OR voice OR acoustic* OR sound* OR microphone* OR “cervical auscultation” OR pressure OR stress OR strain OR stretch* OR accelero* OR vibrat* OR mechanomyo* OR kinematic* OR displacement* OR camera OR optical OR imaging OR image* OR video OR ultraso* OR sonograph* OR sonic OR EMG OR electromyograph* OR wearable OR airflow OR biosignal*) | 16,098,406 |
| #4 | TS=(sensitivity OR specificity OR precision* OR recall OR accuracy OR "positive predictive" OR "negative predictive" OR PPV OR NPV OR AUC OR AUROC OR PR?AUC OR AUC?PR OR "area under curve" OR "receiver operating" OR "diagnostic odds ratio" OR DOR OR F1?score) | 4,631,897 |
| #5 | TS=(paediatric* OR pediatric* OR infant OR birth OR childhood OR children OR “cerebral palsy” OR intubat* OR extubat* OR postextubation OR post-extubation) | 3,123,781 |
| #6 | #1 AND #2 | 7,238 |
| #7 | #6 AND #3 | 3,234 |
| #8 | #7 AND #4 | 623 |
| #9 | #8 NOT #5 | 543 |
| #10 | #8 NOT #5 and Article or Proceeding Paper or Early Access (Document Types) and English (Languages) | 493 |

Search Queries for CINAHL via EbscoHost

| **Number** | **Search Terms** | **Search Hits** |
| --- | --- | --- |
| S1 | Dysphagia OR dysphagic OR "swallowing disorder*" OR "deglutition disorder*" OR “unsafe swallow*” | 15,326 |
| S2 | “deep learning*” OR “machine learning*” OR “neural network*” OR transformer* OR detect* OR monitor* OR classif* OR recogni* OR discriminat* OR distinguish* | 1,022,244 |
| S3 | sensor* OR sensing OR biosens* OR instrument* OR speech OR biomarker* OR voice OR acoustic* OR sound* OR microphone* OR “cervical auscultation” OR pressure OR stress OR strain OR stretch* OR accelero* OR vibrat* OR mechanomyo* OR kinematic* OR displacement* OR camera OR optical OR imaging OR image* OR video OR ultraso* OR sonograph* OR sonic OR EMG OR electromyograph* OR wearable OR airflow OR biosignal* | 1,546,089 |
| S4 | sensitivity OR specificity OR precision* OR recall OR accuracy OR "positive predictive" OR "negative predictive" OR PPV OR NPV OR AUC OR AUROC OR PR?AUC OR AUC?PR OR "area under curve" OR "receiver operating" OR "diagnostic odds ratio" OR DOR OR F1?score | 388,211 |
| S5 | paediatric* OR pediatric* OR infant OR birth OR childhood OR children OR “cerebral palsy” OR intubat* OR extubat* OR postextubation OR post-extubation | 1,183,519 |
| S6 | S1 AND S2 | 2,465 |
| S7 | S6 AND S3 | 1,092 |
| S8 | S7 AND S4 | 184 |
| S9 | S8 NOT S5 | 160 |
| S10 | S9, Source: Academic Journals, Language: English | 156 |

Search Queries for Embase

| **Number** | **Search Terms** | **Search Hits** |
| --- | --- | --- |
| #1 | dysphagia:ti,ab,kw OR dysphagic:ti,ab,kw OR 'swallowing disorder*':ti,ab,kw OR 'deglutition disorder*':ti,ab,kw OR 'unsafe swallow*':ti,ab,kw | 68,609 |
| #2 | 'deep learning*':ti,ab,kw OR 'machine learning*':ti,ab,kw OR 'neural network*':ti,ab,kw OR transformer*:ti,ab,kw OR detect*:ti,ab,kw OR monitor*:ti,ab,kw OR classif*:ti,ab,kw OR recogni*:ti,ab,kw OR discriminat*:ti,ab,kw OR distinguish*:ti,ab,kw | 7,564,978 |
| #3 | sensor*:ti,ab,kw OR sensing:ti,ab,kw OR biosens*:ti,ab,kw OR instrument*:ti,ab,kw OR speech:ti,ab,kw OR biomarker*:ti,ab,kw OR voice:ti,ab,kw OR acoustic*:ti,ab,kw OR sound*:ti,ab,kw OR microphone*:ti,ab,kw OR 'cervical auscultation':ti,ab,kw OR pressure:ti,ab,kw OR stress:ti,ab,kw OR strain:ti,ab,kw OR stretch*:ti,ab,kw OR accelero*:ti,ab,kw OR vibrat*:ti,ab,kw OR mechanomyo*:ti,ab,kw OR kinematic*:ti,ab,kw OR displacement*:ti,ab,kw OR camera:ti,ab,kw OR optical:ti,ab,kw OR imaging:ti,ab,kw OR image*:ti,ab,kw OR video:ti,ab,kw OR ultraso*:ti,ab,kw OR sonograph*:ti,ab,kw OR sonic:ti,ab,kw OR emg:ti,ab,kw OR electromyograph*:ti,ab,kw OR wearable:ti,ab,kw OR airflow:ti,ab,kw OR biosignal*:ti,ab,kw | 8,138,060 |
| #4 | sensitivity:ti,ab,kw OR specificity:ti,ab,kw OR precision*:ti,ab,kw OR recall:ti,ab,kw OR accuracy:ti,ab,kw OR 'positive predictive':ti,ab,kw OR 'negative predictive':ti,ab,kw OR ppv:ti,ab,kw OR npv:ti,ab,kw OR auc:ti,ab,kw OR auroc:ti,ab,kw OR pr?auc:ti,ab,kw OR auc?pr:ti,ab,kw OR 'area under curve':ti,ab,kw OR 'receiver operating':ti,ab,kw OR 'diagnostic odds ratio':ti,ab,kw OR dor:ti,ab,kw OR f1?score:ti,ab,kw | 2,760,055 |
| #5 | paediatric*:ti,ab,kw OR pediatric*:ti,ab,kw OR infant:ti,ab,kw OR birth:ti,ab,kw OR childhood:ti,ab,kw OR children:ti,ab,kw OR 'cerebral palsy':ti,ab,kw OR intubat*:ti,ab,kw OR extubat*:ti,ab,kw OR postextubation:ti,ab,kw OR 'post extubation':ti,ab,kw | 3,003,559 |
| #6 | #1 AND #2 | 14,667 |
| #7 | #6 AND #3 | 6,219 |
| #8 | #7 AND #4 | 839 |
| #9 | #8 NOT #5 | 751 |
| #10 | #9 AND ('article'/it OR 'conference paper'/it) | 363 |
