# Supplementary material for: Current Technological Advances in Dysphagia Screening: Systematic Scoping Review
Source: J Med Internet Res. 2025 May 5;27:e65551. doi: 10.2196/65551 (PMC12089864; doi:10.2196/65551)
Supplement: Multimedia Appendix 4 [file jmir_v27i1e65551_app4.docx]

**Multimedia Appendix 4. Feature extraction, artificial intelligence model, and model training settings in the included studies.**

| **Study** | **Feature Extraction** | **Classifier** | **Train/Test** |
| --- | --- | --- | --- |
| Aboofazeli and Moussavi [35] | PDF: waveform fractal dimension, linear prediction coefficient, average power over frequency bands, RMS of time-domain signal, multi-scale product of wavelet coefficient. | Hidden Markov model | Jackknife CV |
| Basiri et al. [36] | Intrinsic mode functions extracted by AMFB, MFCC, LFBE.  FS: PCA. | SVM (Quadratic) | Leave-one-out |
| Cesarini et al. [37] | PDF: feature set from the Interspeech computational paralinguistic challenge (ComParE) 2016.  FS: Correlation-based selection followed by feature ranking through linear SVM wrapper ranker. | SVM (RBF) | 10-fold CV |
| Donohue et al. [38] | PDF: time, frequency, time-frequency, information-theoretic domains. | SVM, Naïve Baye, logistic regression, decision tree | Leave-one-out |
| Dudik et al. [39] | Multilayer deep belief network (via deep learning) on frequency spectrum. | Multilayer deep belief network | 1700:123 |
| He et al. [40] | PDF: Pitch energy trajectory, Full spectrogram contours, Log symmetric spectral different level, Crucial energy coefficient, Local dynamic features.  FS: t-test, then precise matching analysis. | SVM (Gaussian) | 10-fold CV |
| He et al. [41] | Tensor wheel decomposition to extract band features.  FS: core-pruned tensor wheel. | SVM (linear) | Leave-one-out |
| Inoue et al. [42] | Linear predictive coding. | SVM (RBF) | 10-fold CV |
| Kim et al. [43] | CNN on STFT and MFCC spectrogram (via deep learning). | Multibranch CNN and ensemble model using soft voting | Training-testing ratio different across tasks |
| Lazareck and Moussavi [44] | PDF: time, frequency, time-frequency domains.  FS: t-test. | LDA | Leave-one-out |
| Lee et al. [45] | PDF: time, frequency, time-frequency domains.  FS: genetic algorithm. | LDA, neural network, probabilistic neural network, KNN | 10-fold CV |
| Miyagi et al. [46] | PDF: frequency, time-frequency domains.  FS: correlation. | SVM (RBF) | 27:10 |
| Nikjoo et al. [47] | PDF: time, frequency, information theory-based domains. | SVM on each domain and ensembled using reputation classification | 10-fold CV |
| Ramírez et al. [48] | Soft k-mean clustering. | SVM, AdaBoost, L1-distance model | 3-fold CV |
| Roldan-Vasco et al. [49] | PDF: audio, articulation, diadochokinetic, prosody domains.  FS: t-test. | SVM(linear, RBF, sigmoid), multilayer perceptron, random forest, logistic regression, LDA, decision tree. | Nested CV |
| Roldan-Vasco et al. [50] | PDF: time, frequency, time-frequency, and nonlinear dynamics domains.  FS: min. redundancy max. relevance. | KNN, SVM, ANN, XGboost | 10-fold nested CV |
| Roldan-Vasco et al. [51] | PDF: time domain, frequency domain, time-frequency domain.  FS: min. redundancy max. relevance, PCA, AUC > 0.7. | SVM, ANN, XGBoost | 10-fold/5-fold nested CV |
| Saab et al. [52] | CNN on Mel-spectrogram and three-channel Mel-spectrogram. | DenseNet121, ConvText-Tiny, and ensemble model | 80:20 |
| Sakai et al. [53] | PDF: median, interquartile range of pixel values, number of feature points per pixel estimated from FAST.  FS: Lasso regression with 10-fold CV. | Logistic regression | 70:30 |
| Spadotto et al. [54] | PDF: maximal peak, swallowing apnea period. | SVM (RBF), optimum path forest | CV |
| Spadotto et al. [55] | Feature vectors by the normalized energies of discrete wavelet transform. | Bayesian classification | 10-fold CV |
| Steele et al. [56] | Automatic segmentation and feature extraction (Not specify) | Regularized LDA | 80:20 |
| Wang et al. [57] | PDF: audio domain.  FS: PCA. | Adaboost, SVM, logistic regression | 70:30 |
| Zhao et al. [58] | PDF: audio domain.  FS: PCA. | SVM (RBF), MLP, Adaptive boosting and ensembled model | 75:25 |

ANN: artificial neural network; AMFB: amplitude mean Fourier band; AUC: area under receiver-operating characteristics curve; CNN: convolutional neural network; CV: cross-validation; FAST: features from accelerated segment test; FS: feature selection; KNN: k-nearest-neighbour; LDA: linear discrimination analysis; LFBE: logarithm Fourier band energy; MFCC: Mel-frequency cepstral coefficient; N/A: information not available; PCA: principal component analysis; PDF: predetermined features; RBF: radial basis function; RMS: root mean square; STFT: short-time Fourier transform; SVM: support vector machine; XGBoost; extreme gradient boosting.

35. Aboofazeli M, Moussavi Z. Analysis of swallowing sounds using hidden Markov models. Medical & Biological Engineering & Computing. 2008 Apr;46(4):307-14. PMID: WOS:000254237800001.

36. Basiri B, Vali M, Agah S, Ieee, editors. Classification of Normal and Dysphagia in Patients with GERD Using Swallowing Sound Analysis. 19th CSI International Symposium on Artificial Intelligence and Signal Processing (AISP); 2017 Oct 25-27; Shiraz, IRAN; 2017.

37. Cesarini V, Casiddu N, Porfirione C, Massazza G, Saggio G, Costantini G, et al., editors. A Machine Learning-Based Voice Analysis for the Detection of Dysphagia Biomarkers. IEEE International Workshop on Metrology for Industry 40 & IoT (IEEE MetroInd40 and IoT); 2021 Jun 07-09; Electr Network; 2021.

38. Donohue C, Khalifa Y, Perera S, Sejdić E, Coyle JL. A Preliminary Investigation of Whether HRCA Signals Can Differentiate Between Swallows from Healthy People and Swallows from People with Neurodegenerative Diseases. Dysphagia. 2021 Aug;36(4):635-43. PMID: 32889627.

39. Dudik JM, Coyle JL, El-Jaroudi A, Mao ZH, Sun M, Sejdić E. Deep learning for classification of normal swallows in adults. Neurocomputing. 2018;285:1-9.

40. He F, Hu XY, Zhu C, Li Y, Liu YP. Multi-Scale Spatial and Temporal Speech Associations to Swallowing for Dysphagia Screening. Ieee-Acm Transactions on Audio Speech and Language Processing. 2022;30:2888-99. PMID: WOS:000853834700002.

41. He F, Liu Y, Shen D, Jiang Y, Li Y, Zhu C, editors. Multi-Band Speech Tensor Decomposition for Interactive Feature Extraction in Early Dysphagia Screening. ICASSP 2024-2024 IEEE International Conference on Acoustics, Speech and Signal Processing (ICASSP); 2024: IEEE.

42. Inoue K, Yoshioka M, Yagi N, Nagami S, Oku Y. Using Machine Learning and a Combination of Respiratory Flow, Laryngeal Motion, and Swallowing Sounds to Classify Safe and Unsafe Swallowing. Ieee Transactions on Biomedical Engineering. 2018 Nov;65(11):2529-41. PMID: WOS:000447801800016.

43. Kim H, Park HY, Park D, Im S, Lee S. Non-invasive way to diagnose dysphagia by training deep learning model with voice spectrograms. Biomedical Signal Processing and Control. 2023;86:105259.

44. Lazareck LJ, Moussavi ZM. Classification of normal and dysphagic swallows by acoustical means. IEEE Transactions on Biomedical Engineering. 2004;51(12):2103-12.

45. Lee J, Steele CM, Chau T. Classification of healthy and abnormal swallows based on accelerometry and nasal airflow signals. Artif Intell Med. 2011 May;52(1):17-25. PMID: 21549579.

46. Miyagi S, Sugiyama S, Kozawa K, Moritani S, Sakamoto S, Sakai O. Classifying Dysphagic Swallowing Sounds with Support Vector Machines. Healthcare. 2020 Jun;8(2):103. PMID: WOS:000548056900069.

47. Nikjoo MS, Steele CM, Sejdic E, Chau T. Automatic discrimination between safe and unsafe swallowing using a reputation-based classifier. Biomedical Engineering Online. 2011 Nov;10:100. PMID: WOS:000299213600001.

48. Ramírez J, Rodriquez D, Qiao F, Warchall J, Rye J, Aklile E, et al. Metallic Nanoislands on Graphene for Monitoring Swallowing Activity in Head and Neck Cancer Patients. ACS Nano. 2018 Jun 26;12(6):5913-22. PMID: 29874030.

49. Roldan-Vasco S, Orozco-Duque A, Suarez-Escudero JC, Orozco-Arroyave JR. Machine learning based analysis of speech dimensions in functional oropharyngeal dysphagia. Comput Methods Programs Biomed. 2021 Sep;208:106248. PMID: 34260973.

50. Roldan-Vasco S, Restrepo-Uribe JP, Orozco-Duque A, Suarez-Escudero JC, Orozco-Arroyave JR. Analysis of electrophysiological and mechanical dimensions of swallowing by non-invasive biosignals. Biomedical Signal Processing and Control. 2023 Apr;82:104533. PMID: WOS:000916254200001.

51. Roldan-Vasco S, Orozco-Duque A, Orozco-Arroyave JR. Swallowing disorders analysis using surface EMG biomarkers and classification models. Digital Signal Processing. 2023 Mar;133:103815. PMID: WOS:000922731300011.

52. Saab R, Balachandar A, Mahdi H, Nashnoush E, Perri LX, Waldron AL, et al. Machine-learning assisted swallowing assessment: a deep learning-based quality improvement tool to screen for post-stroke dysphagia. Front Neurosci. 2023;17:1302132. PMID: 38130696.

53. Sakai K, Gilmour S, Hoshino E, Nakayama E, Momosaki R, Sakata N, et al. A Machine Learning-Based Screening Test for Sarcopenic Dysphagia Using Image Recognition. Nutrients. 2021 Nov 10;13(11):4009. PMID: 34836264.

54. Spadotto AA, Pereira JC, Guido RC, Ieee, editors. Oropharyngeal dysphagia identification using wavelets and optimum path forest. 3rd IEEE International Symposium on Control, Communications and Signal Processing (ISCCSP 2008); 2008 Mar 12-14; St Julians, MALTA; 2008.

55. Spadotto AA, Gatto AR, Guido RC, Montagnoli AN, Cola PC, Pereira JC, et al. Classification of normal swallowing and oropharyngeal dysphagia using wavelet. Applied Mathematics and Computation. 2009 Jan;207(1):75-82. PMID: WOS:000262613200006.

56. Steele CM, Mukherjee R, Kortelainen JM, Pölönen H, Jedwab M, Brady SL, et al. Development of a Non-invasive Device for Swallow Screening in Patients at Risk of Oropharyngeal Dysphagia: Results from a Prospective Exploratory Study. Dysphagia. 2019 Oct;34(5):698-707. PMID: WOS:000483701700009.

57. Wang SH, Jiang YY, Zhao HL, Yang X, Zhang ZH, Zhu C, et al., editors. Smart Dysphagia Detection System with Adaptive Boosting Analysis of Throat Signals. IEEE International Symposium on Circuits and Systems (IEEE ISCAS); 2021 May 22-28; Daegu, South Korea; 2021.

58. Zhao HL, Jiang YY, Wang SH, He F, Ren FZ, Zhang ZH, et al. Dysphagia diagnosis system with integrated speech analysis from throat vibration. Expert Systems with Applications. 2022 Oct;204:117496. PMID: WOS:000819313900012.
