# Supplementary material for: Current Technological Advances in Dysphagia Screening: Systematic Scoping Review
Source: J Med Internet Res. 2025 May 5;27:e65551. doi: 10.2196/65551 (PMC12089864; doi:10.2196/65551)
Supplement: Multimedia Appendix 5 [file jmir_v27i1e65551_app5.docx]

**Multimedia Appendix 5. Model performance.**

| **Source** | **Model** | **Study** | **ACC** | **F1** | **AUC** | **Sn** | **Sp** | **Setting remarks^ζ^** |
| --- | --- | --- | --- | --- | --- | --- | --- | --- |
| Sound | HMM | Aboofazeli and Moussavi [35] | 0.855 | - | - | - | - | *N* = 8, Ft: RMS |
| Sound | SVM | Basiri et al. [36] | 0.9565 | - | - | - | - | Ft: IMF extracted from MFCC. |
| Sound | SVM | Cesarini et al. [37]* | 0.9 | - | - | - | - | Consider different feature sets on different tasks. |
| Sound + vibration | LR | Donohue et al. [38] | 0.99 | - | - | 1 | 0.99 | Entire set of features. |
| Vibration | MDBN | Dudik et al. [39] | 0.913 | - | - | 0.857 | 0.949 | Ft: both A-P and S-I, 1^st^ stage: 2 layers, 2^nd^ stage: 1 layer, 3000 neurons per layer . |
| Vibration | SVM | He et al. [40] | 0.892 | - | 0.977 | 0.951 | 0.901 | Tk: reading sentence, ft: LSSDL, C-coes, LDF. |
| Sound | SVM | He et al. [41] | 0.802 | - | 0.874 | 0.85 | 0.804 | Ft: band 2 extracted from C-PTW network. |
| Airflow + biomotion + sound | SVM | Inoue et al. [42] | - | - | - | 0.824 | 0.86 | All modalities, *k* = 32 |
| Sound | CNN | Kim et al. [43] | 0.88 | - | 0.95 | 0.947 | 0.779 | ft: STFT and MFCC |
| Airflow + sound | LDA | Lazareck and Moussavi [44] | - | - | - | 1 | 1 | Tk: thick liquid. |
| Sound | SVM | Lee et al. [45] | 0.780 | 0.781 | - | 0.79 | - | Ft: avg resultant & vertical distances from center of Spm, max magnitude of Spm, frequency median. |
| Strain + EMG | L1 | Miyagi et al. [46] | 0.864 | - | - | - | - | - |
| Sound | EM (SVM, MLP, RF, LR, LDA, DT) | Nikjoo et al. [47] | 0.90 | 0.90 | 0.91 | 0.93 | - | Ft: all features, Tk: all tasks. |
| Vibration + EMG | XGBoost | Ramírez et al. [48] | 0.87 | 0.89 | 0.87 | 0.97 | - | Ft: all modality features, Tk: 5 mL yogurt. |
| EMG | XGBoost | Roldan-Vasco et al. [49] | 0.78 | 0.80 | 0.77 | 0.87 | - | Ft: log-detector, T: 5 mL water. |
| Sound | ConvNext-Tiny | Roldan-Vasco et al. [50] | - | 0.84 | 0.91 | 0.78 | 0.89 | Ft: RGB Mel-spm. |
| Image | LR | Roldan-Vasco et al. [51] | - | - | 0.877 | 0.875 | 0.767 | Ft: age, sex, categorized BMI, median, IQR of pixel value, number of feature points. |
| Sound | OPF | Saab et al. [52] | 0.852 | - | - | - | - | Ft: Max peak of signal |
| Sound | Bayesian | Sakai et al. [53] | 0.76 | - | - | - | - | - |
| Vibration | AdaBoost | Spadotto et al. [54] | 0.712 | - | - | 0.666 | 0.76 | - |
| Vibration | EM (SVM, MLP, AdaBoost) | Spadotto et al. [55] | 0.721 | - | - | 0.636 | 0.810 | *K* = 16 |

*The study reported that all models reached an accuracy over 90% but did not provide the exact values.

^ζ^ all features and all tasks considered by default.

Header Row: ACC: accuracy; AUC: area under receiver-operating characteristics curve; Sn: Sensitivity; Sp: Specificity.

Model Column: CNN: convolutional neural network; DT: decision tree; EM: Ensemble model; LDA: linear discriminant analysis; LR: logistic regression; MDBN: multilayer deep belief network; MLP: multiplayer perceptron; OPF: optimum path finder; RF: random forest; SVM: support vector machine.

Remark Column: A-P: anterior-posterior direction of accelerometers; Avg: average; C-PTW: core-pruned tensor wheel; C-coes: crucial energy coefficient; Ft: feature; IMF: intrinsic mode function; IQR: interquartile range; LDF: local dynamic features; LSSDL: log symmetric spectral difference level; MFCC: Mel-frequency cepstral coefficient; RMS: root mean square; S-I: superior-inferior direction of accelerometers; STFT: short-time Fourier transform; Spm: Spectrogram; Tk: task.

35. Aboofazeli M, Moussavi Z. Analysis of swallowing sounds using hidden Markov models. Medical & Biological Engineering & Computing. 2008 Apr;46(4):307-14. PMID: WOS:000254237800001.

36. Basiri B, Vali M, Agah S, Ieee, editors. Classification of Normal and Dysphagia in Patients with GERD Using Swallowing Sound Analysis. 19th CSI International Symposium on Artificial Intelligence and Signal Processing (AISP); 2017 Oct 25-27; Shiraz, IRAN; 2017.

37. Cesarini V, Casiddu N, Porfirione C, Massazza G, Saggio G, Costantini G, et al., editors. A Machine Learning-Based Voice Analysis for the Detection of Dysphagia Biomarkers. IEEE International Workshop on Metrology for Industry 40 & IoT (IEEE MetroInd40 and IoT); 2021 Jun 07-09; Electr Network; 2021.

38. Donohue C, Khalifa Y, Perera S, Sejdić E, Coyle JL. A Preliminary Investigation of Whether HRCA Signals Can Differentiate Between Swallows from Healthy People and Swallows from People with Neurodegenerative Diseases. Dysphagia. 2021 Aug;36(4):635-43. PMID: 32889627.

39. Dudik JM, Coyle JL, El-Jaroudi A, Mao ZH, Sun M, Sejdić E. Deep learning for classification of normal swallows in adults. Neurocomputing. 2018;285:1-9.

40. He F, Hu XY, Zhu C, Li Y, Liu YP. Multi-Scale Spatial and Temporal Speech Associations to Swallowing for Dysphagia Screening. Ieee-Acm Transactions on Audio Speech and Language Processing. 2022;30:2888-99. PMID: WOS:000853834700002.

41. He F, Liu Y, Shen D, Jiang Y, Li Y, Zhu C, editors. Multi-Band Speech Tensor Decomposition for Interactive Feature Extraction in Early Dysphagia Screening. ICASSP 2024-2024 IEEE International Conference on Acoustics, Speech and Signal Processing (ICASSP); 2024: IEEE.

42. Inoue K, Yoshioka M, Yagi N, Nagami S, Oku Y. Using Machine Learning and a Combination of Respiratory Flow, Laryngeal Motion, and Swallowing Sounds to Classify Safe and Unsafe Swallowing. Ieee Transactions on Biomedical Engineering. 2018 Nov;65(11):2529-41. PMID: WOS:000447801800016.

43. Kim H, Park HY, Park D, Im S, Lee S. Non-invasive way to diagnose dysphagia by training deep learning model with voice spectrograms. Biomedical Signal Processing and Control. 2023;86:105259.

44. Lazareck LJ, Moussavi ZM. Classification of normal and dysphagic swallows by acoustical means. IEEE Transactions on Biomedical Engineering. 2004;51(12):2103-12.

45. Lee J, Steele CM, Chau T. Classification of healthy and abnormal swallows based on accelerometry and nasal airflow signals. Artif Intell Med. 2011 May;52(1):17-25. PMID: 21549579.

46. Miyagi S, Sugiyama S, Kozawa K, Moritani S, Sakamoto S, Sakai O. Classifying Dysphagic Swallowing Sounds with Support Vector Machines. Healthcare. 2020 Jun;8(2):103. PMID: WOS:000548056900069.

47. Nikjoo MS, Steele CM, Sejdic E, Chau T. Automatic discrimination between safe and unsafe swallowing using a reputation-based classifier. Biomedical Engineering Online. 2011 Nov;10:100. PMID: WOS:000299213600001.

48. Ramírez J, Rodriquez D, Qiao F, Warchall J, Rye J, Aklile E, et al. Metallic Nanoislands on Graphene for Monitoring Swallowing Activity in Head and Neck Cancer Patients. ACS Nano. 2018 Jun 26;12(6):5913-22. PMID: 29874030.

49. Roldan-Vasco S, Orozco-Duque A, Suarez-Escudero JC, Orozco-Arroyave JR. Machine learning based analysis of speech dimensions in functional oropharyngeal dysphagia. Comput Methods Programs Biomed. 2021 Sep;208:106248. PMID: 34260973.

50. Roldan-Vasco S, Restrepo-Uribe JP, Orozco-Duque A, Suarez-Escudero JC, Orozco-Arroyave JR. Analysis of electrophysiological and mechanical dimensions of swallowing by non-invasive biosignals. Biomedical Signal Processing and Control. 2023 Apr;82:104533. PMID: WOS:000916254200001.

51. Roldan-Vasco S, Orozco-Duque A, Orozco-Arroyave JR. Swallowing disorders analysis using surface EMG biomarkers and classification models. Digital Signal Processing. 2023 Mar;133:103815. PMID: WOS:000922731300011.

52. Saab R, Balachandar A, Mahdi H, Nashnoush E, Perri LX, Waldron AL, et al. Machine-learning assisted swallowing assessment: a deep learning-based quality improvement tool to screen for post-stroke dysphagia. Front Neurosci. 2023;17:1302132. PMID: 38130696.

53. Sakai K, Gilmour S, Hoshino E, Nakayama E, Momosaki R, Sakata N, et al. A Machine Learning-Based Screening Test for Sarcopenic Dysphagia Using Image Recognition. Nutrients. 2021 Nov 10;13(11):4009. PMID: 34836264.

54. Spadotto AA, Pereira JC, Guido RC, Ieee, editors. Oropharyngeal dysphagia identification using wavelets and optimum path forest. 3rd IEEE International Symposium on Control, Communications and Signal Processing (ISCCSP 2008); 2008 Mar 12-14; St Julians, MALTA; 2008.

55. Spadotto AA, Gatto AR, Guido RC, Montagnoli AN, Cola PC, Pereira JC, et al. Classification of normal swallowing and oropharyngeal dysphagia using wavelet. Applied Mathematics and Computation. 2009 Jan;207(1):75-82. PMID: WOS:000262613200006.
